# Physical activity for insomnia: a scoping review within the Nursing Science Precision Health model

**DOI:** 10.3389/fpubh.2026.1834146

**Published:** 2026-05-28

**Authors:** Xiaotu Zhang, Zihan Qu, Xuefeng Sun, Jiawei Yin, Jingyi Chen, Hongshi Zhang

**Affiliations:** School of Nursing, Changchun University of Chinese Medicine, Changchun, China

**Keywords:** exercise, insomnia, Nursing Science Precision Health model, physical activity, scoping review

## Abstract

**Background:**

Physical activity (PA) is an established non-pharmacological intervention for insomnia; however, its clinical application remains largely non-personalized. The Nursing Science Precision Health (NSPH) model provides a structured framework to link symptom patterns, phenotypes, and biological mechanisms with tailored interventions.

**Objective:**

To map and synthesize evidence on PA interventions for insomnia within the NSPH model, and to identify key gaps limiting precision-based care.

**Methods:**

A scoping review was conducted in accordance with JBI guidance and PRISMA-ScR standards. Eight databases were searched for randomized controlled trials published in the past decade that examined PA interventions for insomnia. Evidence was mapped to the four NSPH domains and synthesized narratively.

**Results:**

Thirty-two studies were included. Across the NSPH domains, three key gaps were identified. First, symptom assessment relied on heterogeneous tools, with no standardized measurement framework. Second, phenotypic characterization focused primarily on psychological and functional outcomes, with limited consideration of lifestyle and environmental factors. Third, although PA demonstrated beneficial effects on sleep quality and related physiological processes, including circadian regulation and inflammatory pathways, biomarker use remained indirect and inconsistently integrated into intervention design. PA interventions, including aerobic, strength, flexibility, and multimodal exercise, generally improved sleep outcomes, but were rarely tailored to individual profiles.

**Conclusion:**

This review highlights the potential of PA interventions for insomnia but emphasizes the need for precision in tailoring treatments to individual patients. Future research should focus on standardizing outcome measures, matching PA modalities to insomnia phenotypes, and incorporating biomarkers to refine and personalize interventions for better patient outcomes.

**Systematic review registration:**

https://doi.org/10.17605/OSF.IO/P4TW6, identifier: P4TW6.

## 1 Introduction

Insomnia is a highly prevalent sleep disorder affecting approximately 30% of adults and is associated with substantial functional impairment, emotional dysregulation, and reduced quality of life (1). From a nursing perspective, it represents a complex, chronic symptom requiring sustained, person-centered management across clinical and community settings (2). Although both pharmacological and non-pharmacological treatments are available, their real-world effectiveness remains limited (3, 4). Pharmacological agents provide short-term relief but are constrained by tolerance, dependence, and adverse effects (5), while recommended first-line behavioral interventions, such as cognitive behavioral therapy for insomnia (CBT-I) (6), relaxation therapy (7), and sleep restriction (8), are often resource-intensive, variably effective, and difficult to sustain in routine practice.

Physical activity (PA), defined as any bodily movement that increases energy expenditure above resting levels, is a promising non-pharmacological intervention for insomnia due to its low cost, feasibility, and integration into daily routines (9). Structured exercise, a subset of PA with defined intensity, frequency, and duration, includes aerobic, resistance, and mind-body modalities such as yoga and Tai Chi. Randomized trials and meta-analyses indicate that both general PA and structured exercise improve subjective sleep quality, reduce insomnia severity, and sometimes enhance CBT-I (10). Emerging evidence suggests that exercise-based interventions, including aerobic exercise, yoga, Tai Chi, and qigong, may benefit insomnia (11); however, formal clinical guidelines do not provide specific recommendations on type, dose, or patient selection (12).

Despite these encouraging findings, the current evidence base for PA interventions in insomnia is not yet well positioned to support precision-oriented care. Interventions vary widely in type, intensity, frequency, duration, and delivery format, and study populations differ substantially in demographic and clinical characteristics (13). The mechanisms linking PA to insomnia outcomes remain incompletely understood, and treatment responses appear highly variable (14). Evidence from chronic yoga interventions further suggests that duration and session frequency may moderate sleep outcomes, indicating that PA responses may vary by both modality and dose (15). Together, these limitations highlight a critical gap: existing research shows that PA can be effective but offers limited guidance for tailoring interventions to individual profiles.

Several theoretical frameworks and models have been applied to guide precision or behaviorally tailored interventions for insomnia, including general behavior change models and symptom-focused precision health approaches. For example, Rajabi Majd et al. (16) applied cognitive behavioral therapy, the Theory of Planned Behavior, the Health Action Process Approach, and Control Theory in an app-based insomnia intervention, while Alkhaldi et al. (17) used the Behavior Change Wheel and COM-B model to address general practitioners' barriers to referring patients with insomnia to Sleepio. Although these models support behavioral change and implementation, they offer limited integration of biological mechanisms, genotypic information, or clinically meaningful insomnia phenotypes into intervention matching (18, 19). Thus, they may only partially address the complexity required for individualized PA interventions.

The Nursing Science Precision Health (NSPH) model, developed by the National Institute of Nursing Research, provides a structured framework for advancing precision-oriented symptom management through four interrelated domains: precise symptom measurement, phenotypic characterization, biomarker discovery, and targeted intervention (20, 21) (Figure shown in Supplementary Appendix A). This model systematically addresses limitations of existing frameworks by integrating symptom, phenotypic, and biological information to guide individualized strategies. Conceptually, NSPH represents a sequential, iterative process in which symptom assessment informs phenotype identification, phenotypic profiles guide exploration of biological mechanisms, and these insights support more targeted interventions.

In PA interventions for insomnia, NSPH directly addresses evidence gaps. Standardized symptom measurement improves comparability, refined phenotypic characterization enables patient stratification, and integration of biomarkers supports mechanistic understanding of treatment effects and variability. This framework is particularly relevant for behavioral interventions where effects are shaped by physiological, behavioral, and contextual factors (22). Despite this alignment, NSPH has rarely been applied to PA interventions for insomnia, and it remains unclear how existing evidence can be reorganized to support individualized care.

Accordingly, this scoping review aims to map and critically examine how PA interventions for insomnia address symptom assessment, phenotypic characterization, biomarker integration, and intervention targeting within the NSPH framework, and to identify key conceptual and methodological gaps that limit translation into precision-oriented care.

## 2 Methods

This scoping review was conducted following the Joanna Briggs Institute (JBI) methodological guidance (23) and reported according to PRISMA-ScR (24). The review protocol was registered on the Open Science Framework (OSF) (https://doi.org/10.17605/OSF.IO/P4TW6).

A scoping review was undertaken due to substantial heterogeneity in PA interventions for insomnia, which limits meta-analysis. We restricted inclusion to randomized controlled trials (RCTs) published over the past decade to enhance rigor. This approach enables mapping of PA interventions across NSPH domains, emphasizing conceptual patterns and evidence gaps rather than effect sizes. Adopting a precision health perspective, this review identifies gaps for future development and offers a novel contribution beyond prior reviews focused primarily on effectiveness.

### 2.1 Research questions

Guided by the NSPH model, this review addressed the following questions:

*Precision in measurement:* How is insomnia assessed in PA intervention trials?

*Precision in phenotype characterization:* What insomnia-related phenotypic characteristics (e.g., demographic/clinical profiles, comorbid symptoms, contextual or lifestyle factors) are reported?

*Precision in the characterization of genotype and other biomarkers:* What genotypic information or biomarkers relevant to insomnia are measured and reported?

*Precision in the identification of intervention targets:* What PA intervention components (type, dose, mode of delivery, and setting) are used to target insomnia symptoms?

### 2.2 Eligibility criteria

Eligibility was defined using the Participants-Concept-Context (PCC) framework. ① *Participants (P):* Individuals with a clinical diagnosis of insomnia, or with poor sleep quality or insomnia symptoms as assessed by validated instruments such as the PSQI, ISI, or AIS. ② *Concept (C):* RCTs evaluating PA interventions for insomnia and reporting insomnia-related outcomes using validated subjective tools and/or objective measures (PSQI, ISI, PSG), with or without biomarker outcomes. ③ *Context (C)*: Any setting in which PA was delivered with the intention of alleviating insomnia symptoms (difficulty initiating sleep, nocturnal awakenings, early morning awakening, or daytime dysfunction).

Exclusion criteria included: ① Studies focusing on primary sleep disorders other than insomnia, or populations with severe medical conditions likely to confound sleep outcomes. ② Non-randomized designs, protocols, conference abstracts, or studies without clearly reported insomnia-related outcomes. ③ Studies published before 2015, to ensure relevance to contemporary PA intervention practices.

### 2.3 Search strategy

Two reviewers independently searched eight sources: CINAHL, Web of Science, Scopus, Embase, Cochrane Library, and the Ovid and ProQuest platforms (platforms providing access to multiple databases). The search covered publications from 20 July 2015 to 20 July 2025 and was limited to English-language studies. Controlled vocabulary (e.g., MeSH terms) and free-text keywords related to insomnia, exercise, and randomized controlled trials were combined. Electronic searches were supplemented with citation tracking and manual screening of reference lists. The full search strategies for all eight sources, including the detailed search string, are provided in Supplementary Appendix B.

### 2.4 Study selection

All records were imported into EndNote X9 for de-duplication. Two reviewers independently screened titles and abstracts, followed by full-text assessment against the eligibility criteria. Discrepancies were resolved through discussion, and unresolved disagreements were adjudicated by a third reviewer. The selection process was summarized in a PRISMA-ScR flow diagram.

### 2.5 Data extraction

Two reviewers independently extracted data using a piloted, standardized form. Extracted items included: ① *Study characteristics*: author(s), year, country/region, journal, design, and sample size. ② *Participant characteristics*: diagnostic criteria, demographic and clinical characteristics, key inclusion/exclusion criteria. ③ *Intervention characteristics*: PA type, intensity, frequency, duration, total dose, supervision/provider, delivery mode, and setting. ④ *Outcomes*: insomnia measures (subjective/objective), timing of assessments, main findings, and reported adverse events where available. ⑤ *Precision health elements*: phenotypic variables, biomarkers (including genotype when reported), and intervention targets relevant to NSPH domains. Extraction discrepancies were resolved by consensus with a third reviewer. If necessary, the senior author provided a final decision. All included studies were cross-checked to ensure consistency and accuracy of extracted information.

### 2.6 Data synthesis

A narrative synthesis was conducted, structured according to the NSPH model. Evidence was mapped across four domains: symptom measurement; phenotypic characterization; biomarker identification; and intervention targeting and delivery. To ensure transparency, predefined coding rules were applied to classify studies across NSPH domains (Table 1). Studies could be coded into multiple domains if criteria were met. Discrepancies in domain assignment were resolved through consensus, with a third reviewer consulted when necessary.

**Table 1:** NSPH coding framework for study classification.

NSPH Domain	Criteria for classification	Examples
Symptom	Assessment of insomnia outcomes using validated subjective or objective measures	PSQI, ISI, sleep onset latency, and nocturnal awakenings
Phenotype	Reporting of individual characteristics relevant to insomnia	Age, anxiety, depression, and comorbid conditions
Biomarker	Measurement of biological or genetic markers related to insomnia	Inflammatory markers and genotype
Intervention	Description and evaluation of PA interventions targeting insomnia	Aerobic exercise, yoga, tai chi, and strength training

### 2.7 Risk of bias assessment

Although critical appraisal is not required for study inclusion in scoping reviews, methodological quality was assessed to support interpretation of findings. Two reviewers independently evaluated risk of bias using the original Cochrane Risk of Bias tool (25), including random sequence generation, allocation concealment, performance bias, detection bias, attrition bias, reporting bias, and other sources of bias. Disagreements were resolved through discussion and consensus.

## 3 Results

### 3.1 Search results

**Figure 1:**
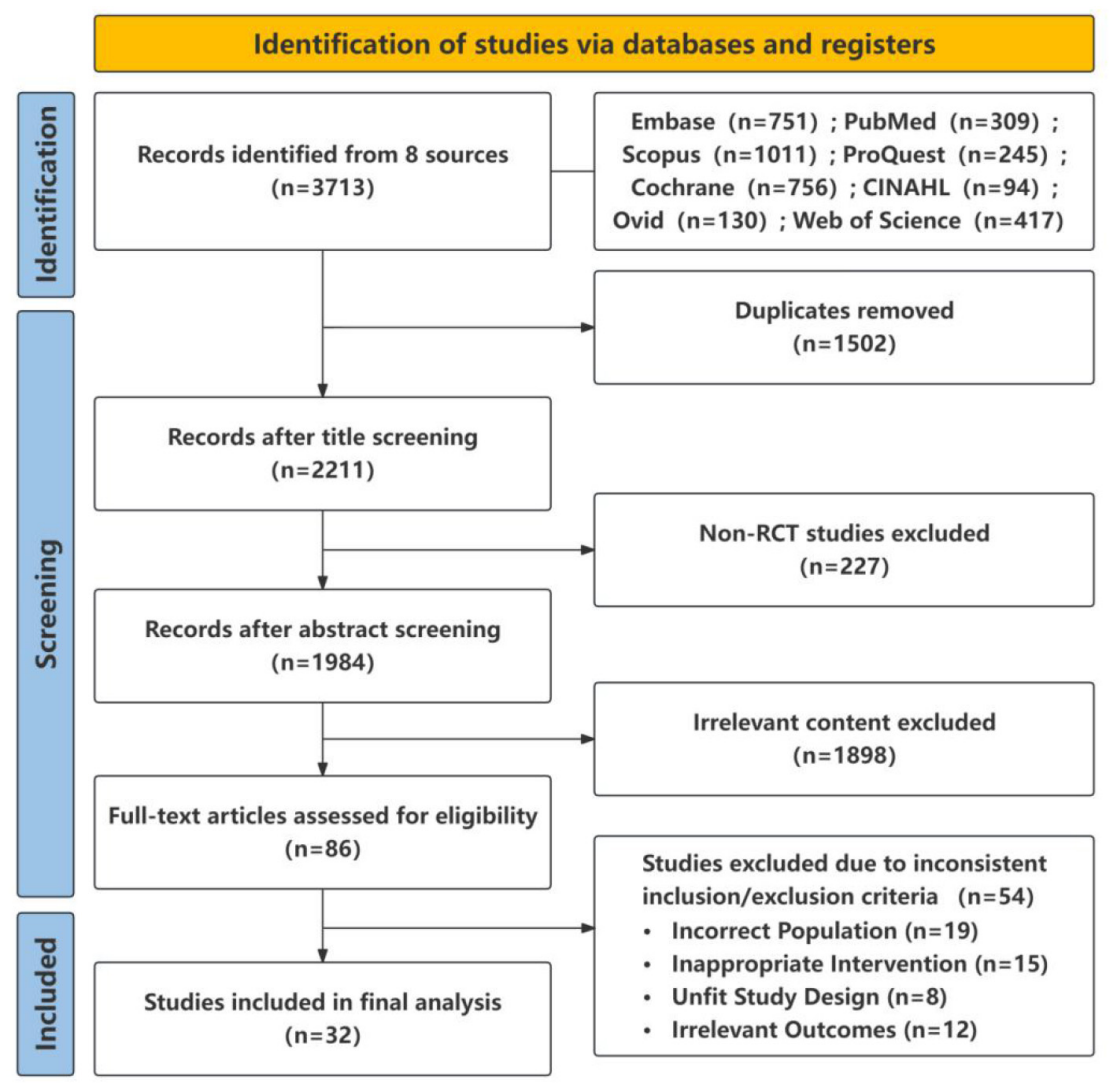
PRISMA flow diagram of the study selection process.

A total of 3,713 records were identified through the database search. After removing duplicates, 2,211 titles and abstracts were screened, leading to the assessment of 86 full-text articles for eligibility. Ultimately, 32 RCTs published between 2015 and 2025 were included in the synthesis (26–57), with the study selection process illustrated in Figure 1.

### 3.2 Risk of bias of included studies

The risk-of-bias assessment showed generally acceptable methodological quality, but with notable uncertainty in several domains (Supplementary Appendix C; Table 2). Most studies were rated as low risk for random sequence generation (78.1%), attrition bias (84.4%), reporting bias (62.5%), and other bias (100.0%). In contrast, allocation concealment was unclear in 50.0% of studies, and detection bias was unclear in 68.8%. Performance bias was the most problematic domain, with 71.9% rated as unclear and 25.0% as high risk, mainly because blinding participants and personnel is difficult in exercise-based interventions. These limitations were considered when interpreting evidence across the NSPH domains.

**Table 2:** Summary of risk-of-bias judgments across included studies.

Risk-of-bias domain	Low risk *n* (%)	Unclear risk *n* (%)	High risk *n* (%)
Random sequence generation	25 (78.1%)	7 (21.9%)	0 (0%)
Allocation concealment	16 (50.0%)	16 (50.0%)	0 (0%)
Performance bias	1 (3.1%)	23 (71.9%)	8 (25.0%)
Detection bias	10 (31.3%)	22 (68.8%)	0 (0%)
Attrition bias	27 (84.4%)	4 (12.5%)	1 (3.1%)
Reporting bias	20 (62.5%)	12 (37.5%)	0 (0%)
Other bias	32 (100.0%)	0 (0%)	0 (0%)

### 3.3 Study characteristics

The 32 included trials were predominantly conducted in Asia, with relatively fewer studies from other regions. Most studies had small-to-medium sample sizes, and only a small number were large-scale trials. Participants were mainly middle-aged and older adults, with limited representation of younger adults. Mixed-sex samples were most common, whereas sex-specific populations were less frequently studied. Overall, the included studies indicate that the current evidence base is geographically concentrated and lacks broad demographic representation (Table 3).

**Table 3:** Study characteristics summary.

Study characteristics		*n*	%
Country	**Asia**	**22**	**68.75**
	·China	14	43.75
	·Iran	5	15.63
	·India	1	3.13
	·Saudi Arabia	2	6.25
	**Europe**	**4**	**12.50**
	·France	1	3.13
	·United Kingdom	2	6.25
	·Spain	1	3.13
	**Americas**	**6**	**18.75**
	·Brazil	4	12.50
	·Canada	1	3.13
	·United States	1	3.13
Average age	**Young adults (18–35)**	**3**	**9.38**
	**Middle-aged adults (36–65)**	**21**	**65.63**
	·36–45	3	9.38
	·46–55	6	18.75
	·56–65	12	37.50
	**Older adults (>65)**	**7**	**21.88**
	·66–75	7	21.88
	·>75	0	0
	**Not reported**	**1**	**3.13**
Sex	·Female	6	18.75
	·Male	3	9.38
	·Both	21	65.63
	·Not reported	2	6.25
Sample size	·1–50	17	53.13
	·51–100	6	18.75
	·101–150	6	18.75
	·151–200	1	3.13
	·201–250	2	6.25

Age categories are based on the mean age of participants in each study.

### 3.4 Mapping evidence onto the NSPH model

**Figure 2:**
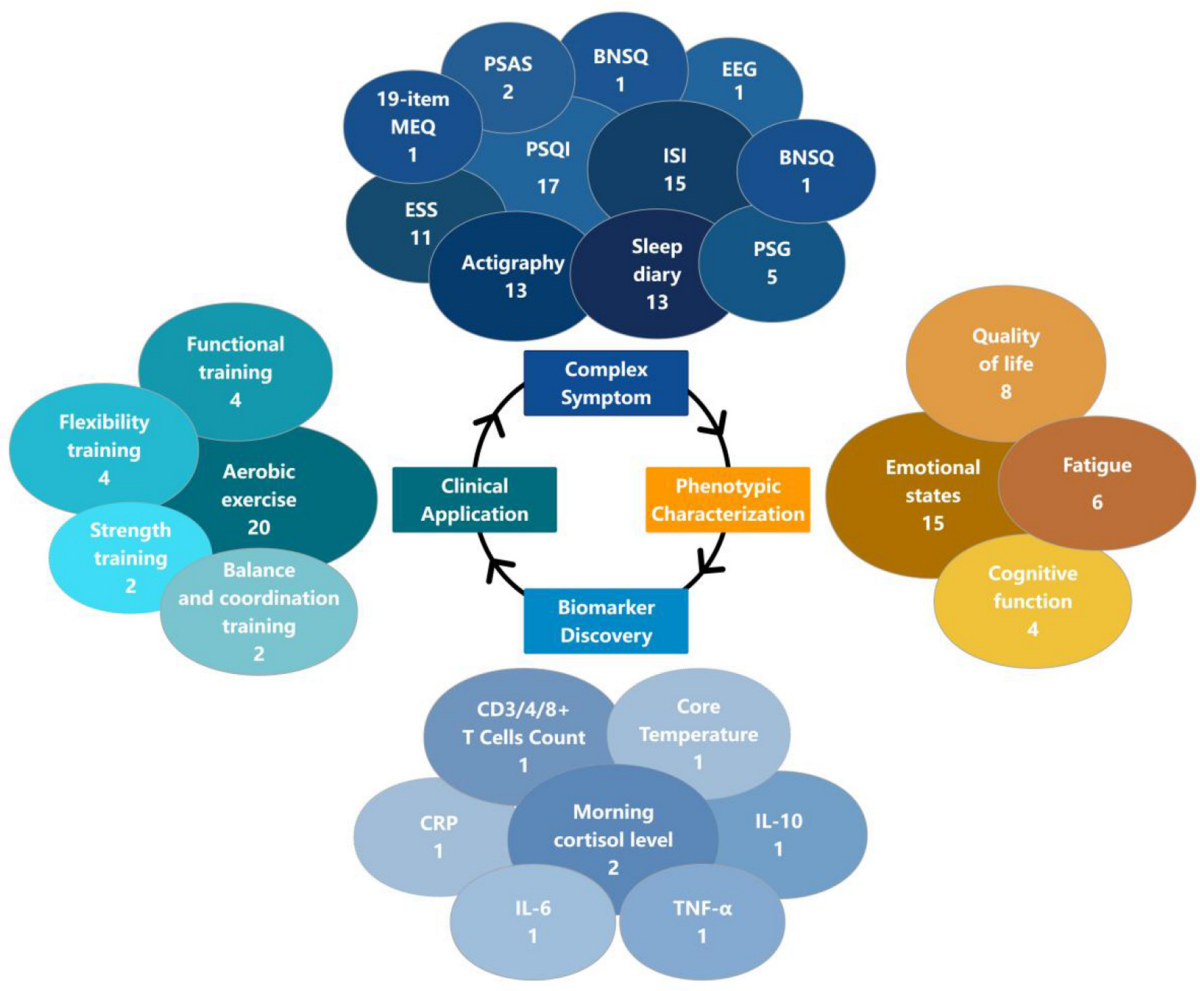
Mapping insomnia and PA onto the NSPH model. The numbers indicate the frequency of studies assessing each domain or intervention. This figure maps physical activity (PA) interventions for insomnia onto the four core components of the Nursing Science Precision Health (NSPH) model, based on the included studies. *Complex symptom assessment* **(top)** highlights the diverse subjective and objective sleep-related measures used (e.g., PSQI, ISI, actigraphy, sleep diary, and PSG). *Phenotypic characterization* **(right)** focuses on emotional states, quality of life, fatigue, and cognitive function. *Biomarker discovery* **(bottom)** reflects the limited and varied use of inflammatory, neuroendocrine, and metabolic markers. *Clinical application* **(left)** summarizes the types of PA interventions, with aerobic exercise being the most commonly studied.

To synthesize the heterogeneity of intervention designs and outcome reporting, findings were organized according to the NSPH model (Figure 2), with detailed study-level mapping provided in Supplementary Appendix D to enhance transparency. Across the four domains, symptom measurement appeared to be the most consistently developed component of the evidence base, while biomarker characterization and intervention contextualization were less developed. Overall, the mapped evidence suggests that while PA research on insomnia has shown potential efficacy, progress has been uneven across the precision-oriented elements necessary for individualized care.

#### 3.4.1 Component 1: precision in measurement

Symptom measurement was the most consistently represented NSPH domain across the included studies. However, this apparent strength was accompanied by substantial heterogeneity in how insomnia was assessed.

For objective sleep outcomes, only a few studies employed PSG or electroencephalography (EEG), likely due to their high cost, operational complexity, and participant burden. PSG, used in 5 studies (26, 27, 30, 32, 37), offers a comprehensive, gold-standard evaluation of sleep architecture. EEG was used in only one study (48) but provides important information on neural correlates of sleep and arousal. Actigraphy (26, 29, 31, 33, 36, 38, 40, 43, 48–50, 54, 56) served as a non-invasive alternative, providing valuable data on sleep-wake cycles, consistency, and efficiency.

For subjective sleep outcomes, most studies employed self-reported instruments to assess sleep experience and symptom perception, although the measurement dimensions and sensitivity varied substantially. PSQI, used in 17 studies (28–31, 33–35, 41, 45, 46, 50–55, 57), was the most commonly applied subjective tool, followed by the ISI (27–30, 38, 43, 44, 46–50, 54–56), both of which are critical for assessing sleep quality and severity. The Sleep Diary (26, 28–30, 42, 44, 47–51, 54, 56) provided detailed insights into daily sleep patterns, while tools like the Epworth Sleepiness Scale (ESS) (27, 28, 38, 41, 43–46, 49, 55, 57) and the 19-item Morningness-Eveningness Questionnaire (MEQ) (33) were employed to assess daytime sleepiness and chronotype, respectively. The Basic Nordic Sleep Questionnaire (BNSQ) (42) was applied to evaluate general sleep patterns and common disturbances, while the Rapid Eye Movement Sleep Behavior Disorder Scale (RBDS) served as a screening tool for REM sleep behavior disorder. To capture arousal mechanisms relevant to insomnia, the Pre-Sleep Arousal Scale (PSAS) (48, 49) measured cognitive and somatic arousal at bedtime.

#### 3.4.2 Component 2: precision in phenotype

Phenotypic characterization was less developed and more inconsistently operationalized than symptom measurement. Of the 32 studies reviewed, 19 studies (27–32, 34, 38, 43, 44, 46, 48, 49, 52–57) examined the relationship between phenotypic traits and insomnia. The analysis of phenotypic characteristics in individuals with insomnia reveals that most studies focused on the associations between phenotype clusters and insomnia, with several examining the impact of multiple emotional and physiological symptoms, such as anxiety, depression, stress, fatigue, and cognitive dysfunction. However, only 3 studies (32, 34, 57) specifically analyzed the impact of a single symptom or trait. Among these 19 studies, 15 studies (28–32, 38, 43, 46, 48, 49, 52–56) investigated the relationship between insomnia and emotional disorders such as anxiety, depression, and stress, emphasizing the significant role of emotional disorders in the onset and progression of insomnia. Furthermore, 8 studies (30, 38, 43, 48, 49, 52, 54, 56) examined the impact of insomnia on quality of life, revealing significant impairments in social functioning and daily activities. 4 studies (34, 52, 53, 57) focused on the adverse effects of insomnia on cognitive function, indicating that chronic insomnia may lead to cognitive decline, particularly in attention, memory, and executive function. Another 6 studies (38, 43–45, 54, 56) explored the relationship between insomnia and fatigue, identifying insomnia as a major contributor to fatigue, which exacerbates the vicious cycle of insomnia. Some phenotypic traits, such as stress, health perception, and self-esteem, have also been addressed in insomnia research, although they were less frequently studied.

At the same time, phenotypic evidence remained largely descriptive. Most studies identified co-occurring characteristics of insomnia, but few translated these characteristics into meaningful clinical subgrouping or intervention differentiation. Lifestyle and contextual dimensions were especially underdeveloped, despite their likely relevance to behavioral intervention response. Overall, the literature reflects growing awareness of phenotypic complexity, but not yet a mature precision-based use of phenotype to inform who should receive which type of PA intervention.

#### 3.4.3 Component 3: precision in the characterization of genotype and other biomarkers

Biomarker evidence was limited and remained one of the least developed domains in the current literature. Only 8 trials (26, 28, 30–32, 37, 43, 51) reported biomarker outcomes following PA interventions, and no study included genotype-related measures (Table 4).

**Table 4:** Biomarker outcomes of PA interventions in insomnia.

Author/Year	Exercise group	Control group	Biomarker changes	Clinical implications
Rozales et al. (26)	**Acute aerobic exercise (treadmill)** 50% HRR ±5 bpm, single session	**Acute zolpidem intake** 10 mg at bedtime	Morning cortisol ↓	Cortisol decreased after exercise; no significant between-group difference (*p* > 0.05).
Ferreira et al. (30)	**Aerobic exercise (treadmill)** 50% HRR ±5 bpm, 3x/week, 50 min/session, 12 weeks	**Aerobic exercise+acupuncture** 2x/week, 50 min/session, 12 weeks	Morning cortisol ↓	Cortisol reduction was moderately correlated with ISI improvement (r = 0.56, *p* < 0.05).
Abd et al. (32)	**Aerobic exercise (treadmill)** 60–70% maxHF, 3x/week, 45 min/session, 6 months	**Usual lifestyle**	CD3^+^ counts ↓ CD4^+^ counts ↓ CD8^+^ counts ↓	cell counts decreased; CD4^+^/CD8^+^ ratio remained stable (*p* < 0.05).
El-Kader et al. (37)	**Aerobic exercise (treadmill)** 60–80% maxHF, 3x/week, 40 min/session, 6 months	**Usual lifestyle**	IL-6 ↓, TNF-α ↓, IL-10 ↑	Pro-inflammatory cytokines (IL-6 and TNF-α) decreased and IL-10 increased (*p* < 0.05).
Judith et al. (51)	**Flexibility exercise (Tai Chi)** low–moderate intensity, 1x/week, 120 min/session, 4 months	**Sleep seminar** 1x/week, 120 min/session, 4 months	HDL ↑ LDL ↓ TG ↓ Fibrinogen ↓ CRP ↓ Glucose ↓ Insulin ↓ HbA1c ↓	Lipid, glycaemic and inflammatory markers improved (all *p* < 0.05).
Baron et al. (27)	**Aerobic exercise (walking/treadmill)** 70% maxHF (50 min), >80% maxHF (25 min), 3x/week, 75 min/session, 12 weeks	**Usual lifestyle**	Core body temperature: Amplitude ↑ Batyphase (minimum temperature)↓	Temperature rhythm indicators changed and were correlated with insomnia symptom improvement (*p* < 0.05).
Iuliana et al. (43)	**Aerobic exercise (brisk walking)** moderate intensity, 5x/week, ≥30 min/session, 6 months	**Usual lifestyle**	BMI ↓	BMI decreased, sleep onset latency shortened, and depressive symptoms alleviated (*p* < 0.05).
Tseng et al. (31)	**Aerobic exercise (treadmill walking and stretching)** 40–60% VO_2_peak, 3x/week, 50 min/session, 12 weeks	**Usual lifestyle**	HRV: LF ↓; HF ↑; LF/HF ↓	Autonomic function improved (*p* < 0.05).

IL-6, interleukin-6; IL-10, interleukin-10; TNF-α, tumor necrosis factor-α; LDL, low-density lipoprotein; HDL, high-density lipoprotein; TG, triglycerides; CRP, C-reactive protein; BMI, body mass index; HRV, heart-rate variability; LF, low frequency; HF, high frequency; HRR, heart-rate reserve; maxHF, maximal heart rate.

Findings showed that acute exercise reduced cortisol without significant between-group differences (26), while long-term exercise showed a moderate correlation between cortisol reduction and insomnia improvement(30), suggesting a cumulative therapeutic effect. Regular exercise was linked to reduced CRP, IL-6, TNF-α, and CD3^+^, CD4^+^, and CD8^+^ T-cell counts (32), alongside increased anti-inflammatory IL-10 (37), reducing the inflammatory risk phenotype in insomnia. Metabolically, exercise lowered BMI, adjusted circadian body temperature rhythms, and improved autonomic function (28). Tseng et al. found that 12 weeks of moderate-intensity aerobic exercise increased HF and decreased LF and LF/HF ratios, reflecting parasympathetic dominance, with HRV improvements associated with better sleep quality (31). Similarly, Iuliana et al. (43) reported that aerobic walking reduced BMI, shortened sleep onset latency, and alleviated depressive symptoms, suggesting interactions between metabolic improvements and sleep restoration. Additionally, Judith et al. demonstrated that Tai Chi improved sleep quality and provided systemic benefits, including better lipid, glycemic, and inflammatory profiles, highlighting the holistic health effects of PA interventions in insomnia management (51).

Despite these signals, biomarker assessment remained highly heterogeneous in both indicator selection and study design, and most findings were derived from a small number of studies. More importantly, biomarkers were used primarily as exploratory mechanistic outcomes rather than as actionable variables for patient stratification, intervention matching, or treatment optimization. Thus, while the current evidence provides preliminary biological support for PA-based interventions, this NSPH domain remains insufficiently developed to inform truly individualized care.

#### 3.4.4 Component 4: precision in the identification of intervention targets

Most studies employed RCT designs, with the majority using two-arm comparisons of PA interventions vs. control conditions, and some adopting multi-group designs to compare different exercise modalities or combine PA with psychological or educational approaches. Detailed descriptions of study designs, intervention types, and delivery characteristics are provided in Supplementary Appendix E.

Aerobic exercise was the most frequently applied intervention (62.50%) (26–45), encompassing activities such as treadmill walking, jogging, brisk walking, cycling, and aquatic-based walking or swimming. Functional exercise (12.50%) (54–57), which integrates stretching, coordination, balance, and light resistance exercises, was the second most common approach. Flexibility exercise (12.50%) (48–51), such as Tai Chi (8-, 12-, or 24-form), yoga, and stretching, were also reported. Strength exercise accounted for 6.25% of interventions (46, 47), while balance and coordination exercise was described in 6.25% of studies (52, 53).

Intervention frequency ranged from two to five sessions per week, with session durations of 40–75 min and intervention periods spanning 8–24 weeks. Some studies assessed acute effects following a single bout of exercise (26, 36), whereas others extended intervention delivery to 6–12 months (32, 37, 38, 42, 43). Control conditions most often involved usual lifestyle maintenance, although some included relaxation therapy (28), sleep hygiene education (31, 54, 56, 57), or pharmacological treatment (26).

PA interventions generally showed beneficial effects on insomnia-related outcomes, particularly in reducing insomnia severity, improving sleep quality, shortening sleep onset latency, increasing sleep efficiency, and improving daytime symptoms. However, findings were less consistent for objective sleep parameters such as total sleep time, wake after sleep onset, sleep architecture, and actigraphy-derived outcomes, and between-group effects were not always statistically significant. The heterogeneity of PA modality, dose, outcome measures, and follow-up timing further limits direct comparison across studies. Detailed sleep-related outcomes, including intervention-group effects, between-group differences, reported *p*-values, and directions of effect, are summarized in Supplementary Appendix F.

Provider and setting characteristics were inconsistently reported. 37.50% of interventions were delivered by exercise or rehabilitation professionals (27–29, 31–34, 36, 39–41, 47), while nurses (41, 54, 56), mental health providers (44, 47), and traditional exercise instructors (48–51) were less frequently specified. Notably, over one-third of the studies did not clearly describe provider qualifications. Indoor treadmill-based exercise (26, 28–30, 32, 33, 36, 38, 39) was most frequently reported, followed by home-based programs (27, 47, 49, 54–57), medical institutions (31, 34, 41, 42, 52), and community-based programs (27, 40, 48). However, one-fourth of studies did not report the intervention setting, limiting the assessment of contextual precision. Details are provided in Table 5.

**Table 5:** PA intervention characteristics.

Intervention characteristics		*n*	%
Intervention method	Aerobic exercise	20	62.50
	Functional exercise	4	12.50
	Flexibility exercise	4	12.50
	Balance and coordination exercise	2	6.25
	Strength exercise	2	6.25
Intervention delivery	Exercise/Rehabilitation professionals	12	37.50
	Mental health professionals	2	6.25
	Nurses/Medical staff	3	9.38
	Traditional exercise instructors	4	12.50
	NA	11	34.38
Intervention settings	Medical institution	5	15.63
	Community	3	9.38
	Home	7	21.88
	Indoor treadmill setting	9	28.13
	NA	8	25.00

## 4 Discussion

### 4.1 Summary of findings and alignment with the NSPH model

This scoping review underscores the significant role of PA in improving sleep outcomes in individuals with insomnia. Numerous studies have consistently demonstrated that PA not only enhances sleep quality but also reduces insomnia severity, shortens sleep onset latency, and improves daytime functioning. Additionally, PA has shown positive effects on comorbid psychological symptoms, including anxiety and depression, further supporting its multidimensional therapeutic potential. However, despite the documented benefits, most research has not adequately addressed how to tailor these interventions to individual patient profiles, which is a central tenet of the NSPH model.

While current studies predominantly focus on generalized efficacy, they often overlook the personalized factors, such as age, gender, psychological profile, and lifestyle, that could mediate the response to PA interventions (58). This review emphasizes the critical need for future research that aligns with the NSPH model's principles, integrating phenotypic characterization and biomarker profiling to enhance the precision of PA interventions for insomnia.

### 4.2 Challenges in precision measurement and recommendations

A major challenge in PA interventions for insomnia is the lack of standardized symptom measurement. Although validated tools like PSQI and ISI are commonly used, their varying dimensions and assessment timing create inconsistencies across studies, limiting comparability and generalizability (59). While PSG and EEG are gold-standard measures, their high cost and complexity make them impractical for widespread clinical use (60). Actigraphy, a more feasible alternative, is often used in isolation, limiting its ability to capture a comprehensive picture of sleep disturbances (61). Furthermore, discrepancies between subjective evaluations and objective recordings highlight the complex interplay between physiological regulation and psychological perception, yet few studies integrate multidimensional frameworks assessing psychological, physiological, and quality-of-life outcomes (62).

To address these challenges, future research should prioritize the development of a standardized, multidimensional measurement framework that integrates both subjective and objective sleep assessments. This would provide a more nuanced understanding of sleep disturbances and better support the design of individualized treatment strategies. Additionally, incorporating wearable technologies to monitor physiological parameters continuously, combined with subjective sleep reports, could provide real-time insights, enabling adaptive, precision-based interventions (63).

### 4.3 Insufficient phenotypic characterization and future directions

Although phenotypic variability in insomnia is increasingly recognized, current studies remain largely descriptive and rarely link phenotypic traits to PA intervention outcomes. Emotional distress, cognitive dysfunction, and fatigue are frequently reported in insomnia, and psychological factors such as anxiety and depression may influence PA responsiveness (64, 65). However, evidence remains limited on how these characteristics mediate or moderate improvements in insomnia. Response variability is also evident within a single PA modality; for example, a recent scoping review of chronic stretch training found that only 5 of 16 studies reported significant sleep improvements, despite an overall positive trend (66). This unexplained variability highlights the need to link individual phenotypes, biomarkers, and contextual profiles with intervention matching.

To bridge this gap, future studies should implement more rigorous phenotypic stratification, considering not only psychological but also physiological factors. For instance, age, sex, and lifestyle behaviors (e.g., physical activity habits, sleep environment) should be integrated into phenotypic assessments (67). This will allow for a more nuanced understanding of how different phenotypic profiles respond to PA interventions and guide the design of targeted treatment plans.

### 4.4 Potential of biomarkers and integration into precision care

Biomarkers hold considerable promise in enhancing the precision of PA interventions for insomnia. Research has shown that PA can modulate inflammatory markers (e.g., IL-6, TNF-α), cortisol levels, and autonomic function, which are all implicated in the pathophysiology of insomnia (68). Despite these findings, biomarkers are typically used as secondary outcomes in most studies, limiting their integration into intervention design and patient stratification. As a result, the potential of biomarkers to guide precision-based interventions has not been fully realized.

To optimize PA interventions, future research should integrate biomarkers such as inflammatory markers and autonomic function indicators as primary variables in study designs. These biomarkers could serve as key tools for identifying suitable patient subgroups, thereby enhancing the efficacy of PA interventions (69). Furthermore, the use of digital health tools, including wearable devices, to monitor real-time physiological data presents an opportunity to combine biomarker assessments with continuous monitoring, allowing for adaptive and personalized care (70). This integration would play a pivotal role in transforming PA interventions into more targeted, biologically informed treatments for insomnia.

### 4.5 Nurse-led PA interventions: unleashing their potential

Despite the well-established role of nurses in behavioral support, patient education, and long-term symptom management, nurse-led PA interventions for insomnia remain virtually untested. In this review, only a small number of studies reported nurse or medical staff involvement in intervention delivery, whereas most interventions were led by exercise therapists, rehabilitation professionals, or other providers. Given nurses' central role in chronic symptom management and sustained patient engagement, this represents a high-priority research gap (71).

Future research should explore nurse-led models of PA intervention, particularly in community and home care settings, where nurses' ability to provide holistic care can be fully leveraged (72). By enhancing nurses‘ training in PA interventions and incorporating them into clinical treatment pathways, we can improve intervention adherence, sustainability, and effectiveness, especially in resource-limited settings.

### 4.6 Proposed theoretical extension of the NSPH model for behavioral precision

The findings of this review suggest that the NSPH model provides a useful structure for organizing evidence on PA interventions for insomnia across symptom measurement, phenotypic characterization, biomarker assessment, and intervention targeting. However, the included studies also showed that behavioral intervention characteristics, including PA modality, intensity, timing, adherence, delivery context, and patient preferences, were not consistently incorporated into precision-oriented intervention design or linked to differential treatment responses.

Based on these evidence gaps, we propose a theoretical extension of the NSPH model rather than an empirical finding derived directly from the included trials. Future applications of the NSPH model may benefit from adding a behavioral precision layer that links symptom profiles, phenotypes, biomarkers, and contextual factors to specific PA prescriptions, including modality, dose, timing, and delivery format (73). In future studies, real-time feedback through wearable monitoring of sleep, activity, and physiological responses could be explored as a potential strategy to support adaptive intervention adjustment (74), but this remains a conceptual direction requiring empirical testing in well-designed prospective studies.

### 4.7 Limitations and future research directions

Several limitations warrant caution. First, the majority of included studies had small sample sizes and short intervention or follow-up periods, limiting conclusions regarding long-term efficacy. Second, more than half did not specify the qualifications of intervention providers or the setting, reducing reproducibility and applicability. Third, very few studies incorporated biomarkers, genetic data, or comprehensive phenotyping, preventing deeper insights into underlying mechanisms. Fourth, geographic concentration in Asia raises concerns about cultural generalizability, as exercise behaviors and perceptions of insomnia differ across regions. Finally, the restriction to English-language publications may have introduced language bias and led to the exclusion of relevant evidence published in other languages.

Future research should move beyond demonstrating efficacy toward developing precision-oriented intervention frameworks. This includes conducting large-scale, multicenter trials with standardized measurement protocols, integrating multidimensional phenotyping and biomarker assessment into study design, and leveraging digital health technologies to capture real-time behavioral and physiological data. Importantly, greater emphasis should be placed on nurse-led intervention models to enhance scalability and translation into practice. Strengthening these areas will be essential for advancing PA interventions from broadly effective approaches to truly individualized strategies for insomnia care.

## 5 Conclusion

This review highlights the potential of PA interventions for insomnia while emphasizing the need for precision in tailoring treatments to individual patients. Future research should focus on developing standardized outcome measures for PA-insomnia trials, designing stratified trials that match PA modalities to specific insomnia phenotypes, and integrating biomarkers into study designs to refine and personalize interventions, ultimately ensuring more effective treatments for insomnia.

## Data Availability

The original contributions presented in the study are included in the article/Supplementary material, further inquiries can be directed to the corresponding authors.
