# Supplementary figures and images for: Physical activity for insomnia: a scoping review within the Nursing Science Precision Health model
Source: Front Public Health. 2026 May 28;14:1834146. doi: 10.3389/fpubh.2026.1834146 (PMC13253286; doi:10.3389/fpubh.2026.1834146)

## Appendix A: Schematic Diagram of the NSPH Model

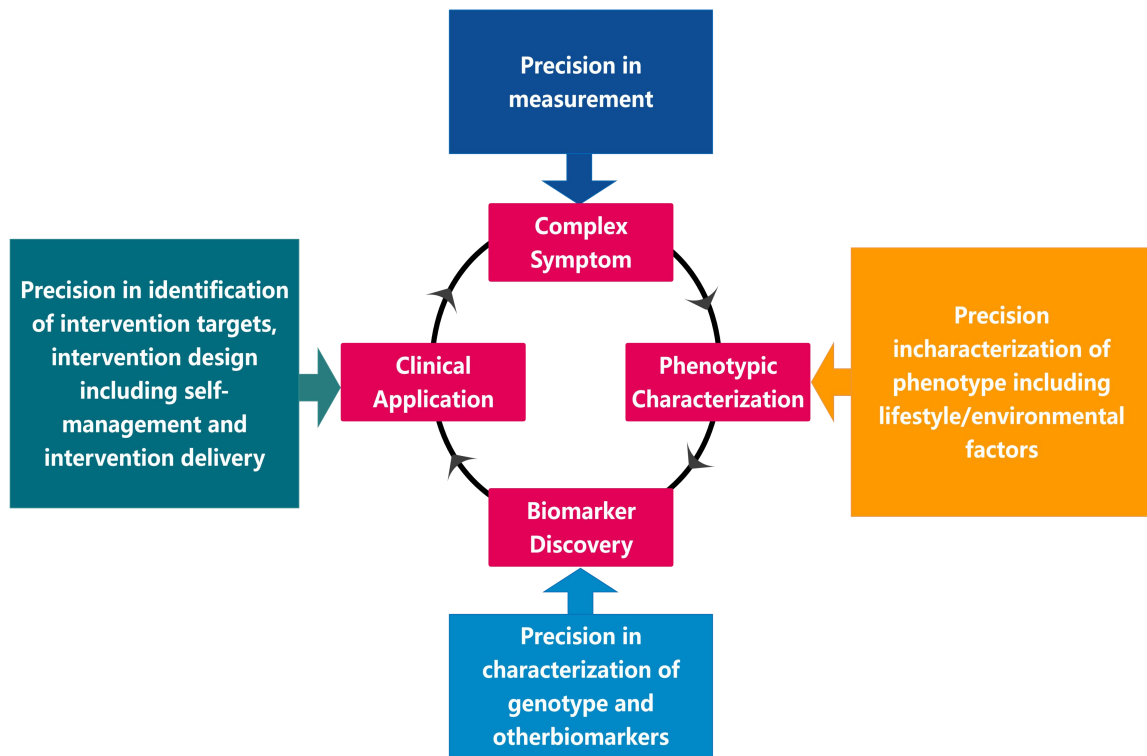

Supplement: Supplementary file 1 [file Supplementary_File_1.pdf]
