# Supplementary material for: Physical activity for insomnia: a scoping review within the Nursing Science Precision Health model
Source: Front Public Health. 2026 May 28;14:1834146. doi: 10.3389/fpubh.2026.1834146 (PMC13253286; doi:10.3389/fpubh.2026.1834146)
Supplement: Supplementary file 2 [file Supplementary_File_2.pdf]

Appendix B：Search Words and Search Strategy

➢ Search Words

1. *“Physical activity” search words*

MeSH word:

- Exercise

Entry Terms:

- Exercises
- Exercise, Physical
- Exercises, Physical
- Physical Exercise
- Physical Exercises
- Exercise, Aerobic
- Aerobic Exercise
- Aerobic Exercises
- Exercises, Aerobic
- Exercise, Isometric
- Exercises, Isometric
- Isometric Exercises
- Isometric Exercise
- Acute Exercise
- Acute Exercises
- Exercise, Acute
- Exercises, Acute
- Exercise Training
- Exercise Trainings
- Training, Exercise
- Trainings, Exercise
- Physical Activity
- Activities, Physical
- Activity, Physical
- Physical Activities
- Traditional exercise
- Exercises of Chinese medicine

- TCM exercise
- Tai Ji
- Tai-ji
- Tai Chi
- Chi, Tai
- Tai Ji Quan
- Ji Quan, Tai
- Quan, Tai Ji
- Taiji
- Taijiquan
- T'ai Chi
- Tai Chi Chuan
- Qigong
- Qi Gong
- Ch'i Kung
- Baduanjin
- wuqinxi
- Five-animal play
- Five-animal exercise
- LQG
- Liuzijue
- Six character formula
- Six character code
- Six character pithy
- Yi Jin Jing

2. *“Insomnia” search words*

MeSH word:

- Sleep Initiation and Maintenance Disorders

Entry Terms:

- DIMS (Disorders of Initiating and Maintaining Sleep)
- Disorders of Initiating and Maintaining Sleep
- Sleeplessness
- Insomnia Disorder
- Insomnia Disorders

- Insomnia
- Insomnias
- Chronic Insomnia
- Insomnia, Chronic
- Early Awakening
- Awakening, Early
- Nonorganic Insomnia
- Insomnia, Nonorganic
- Primary Insomnia
- Insomnia, Primary
- Psychophysiological Insomnia
- Insomnia, Psychophysiological
- Rebound Insomnia
- Insomnia, Rebound
- Secondary Insomnia
- Insomnia, Secondary
- Sleep Initiation Dysfunction
- Dysfunction, Sleep Initiation
- Dysfunctions, Sleep Initiation
- Sleep Initiation Dysfunctions
- Transient Insomnia
- Insomnia, Transient

3. *"Randomized Controlled Trial" search words*

MeSH word:

- Randomized Controlled Trial

Entry Terms:

- Randomised
- Randomized
- Placebo
- RCT
- Random

➢ Search Strategy

1. Cochrane-756

#1	((Exercise) OR (Exercises) OR (Exercise, Physical) OR (Exercises, Physical) OR (Physical Exercise) OR (Physical Exercises) OR (Exercise, Aerobic) OR (Aerobic Exercise) OR (Aerobic Exercises) OR (Exercises, Aerobic) OR (Exercise, Isometric) OR (Exercises, Isometric) OR (Isometric Exercises) OR (Isometric Exercise) OR (Acute Exercise) OR (Acute Exercises) OR (Exercise, Acute) OR (Exercises, Acute) OR (Exercise Training) OR (Exercise Trainings) OR (Training, Exercise) OR (Trainings, Exercise) OR (Physical Activity) OR (Activities, Physical) OR (Activity, Physical) OR (Physical Activities) OR (Traditional exercise) OR (Exercises of Chinese medicine) OR (TCM exercise) OR (Tai Ji) OR (Tai-ji) OR (Tai Chi) OR (Chi, Tai) OR (Tai Ji Quan) OR (Ji Quan, Tai) OR (Quan, Tai Ji) OR (Taiji) OR (Taijiquan) OR (T'ai Chi) OR (Tai Chi Chuan) OR (Qigong) OR (Qi Gong) OR (Ch'i Kung) OR (Baduanjin) OR (wuqinxi) OR (Five-animal play) OR (Five-animal exercise) OR (LQG) OR (Liuzijue) OR (Six character formula) OR (Six character code) OR (Six character pithy) OR (Yi Jin Jing)):ab,ti
#2	((Sleep Initiation and Maintenance Disorders) OR (DIMS (Disorders of Initiating and Maintaining Sleep)) OR (Disorders of Initiating and Maintaining Sleep) OR (Sleeplessness) OR (Insomnia Disorder) OR (Insomnia Disorders) OR (Insomnia) OR (Insomnias) OR (Chronic Insomnia) OR (Insomnia, Chronic) OR (Early Awakening) OR (Awakening, Early) OR (Nonorganic Insomnia) OR (Insomnia, Nonorganic) OR (Primary Insomnia) OR (Insomnia, Primary) OR (Psychophysiological Insomnia) OR (Insomnia, Psychophysiological) OR (Rebound Insomnia) OR (Insomnia, Rebound) OR (Secondary Insomnia) OR (Insomnia, Secondary) OR (Sleep Initiation Dysfunction) OR (Dysfunction, Sleep Initiation) OR (Dysfunctions, Sleep Initiation) OR (Sleep Initiation Dysfunctions) OR (Transient Insomnia) OR (Insomnia, Transient)):ab,ti
#3	((Randomized Controlled Trial) OR (Randomised) OR (Randomized) OR (Placebo) OR (RCT) OR (Random)):ab,ti
#4	#1 AND #2 AND #3

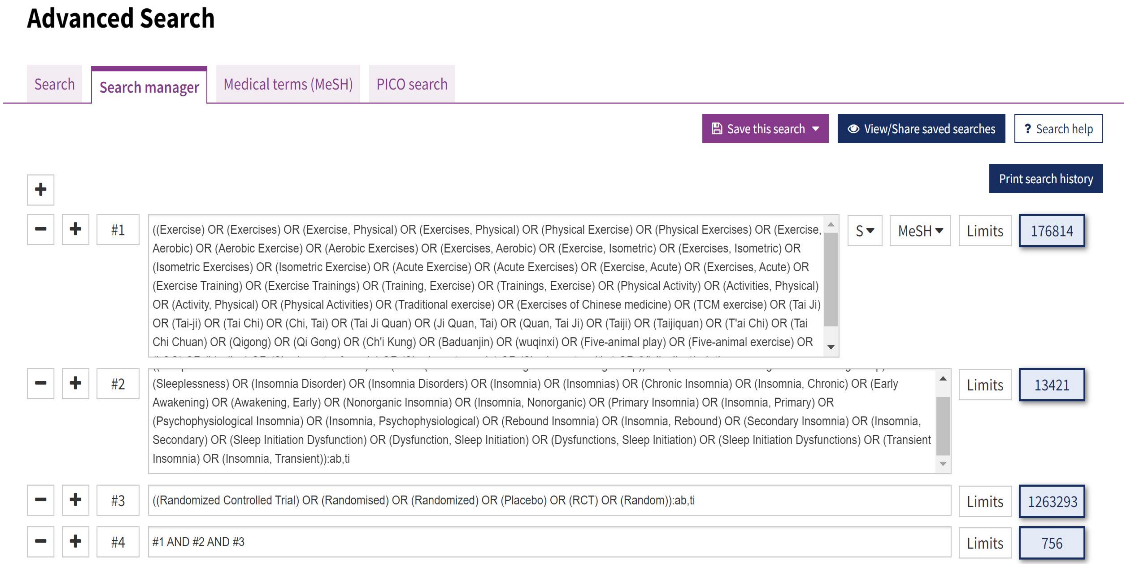

2. Web of science-417

#1	AB,TI=(Sleep Initiation and Maintenance Disorders OR DIMS (Disorders of Initiating and Maintaining Sleep) OR Disorders of Initiating and Maintaining Sleep OR Sleeplessness OR Insomnia Disorder OR Insomnia Disorders OR Insomnia OR Insomnias OR Chronic Insomnia OR Insomnia, Chronic OR Early Awakening OR Awakening, Early OR Nonorganic Insomnia OR Insomnia, Nonorganic OR Primary Insomnia OR Insomnia, Primary OR Psychophysiological Insomnia OR Insomnia, Psychophysiological OR Rebound Insomnia OR Insomnia, Rebound OR Secondary Insomnia OR Insomnia, Secondary OR Sleep Initiation Dysfunction OR Dysfunction, Sleep Initiation OR Dysfunctions, Sleep Initiation OR Sleep Initiation Dysfunctions OR Transient Insomnia OR Insomnia, Transient)
#2	AB,TI=(Exercise OR Exercises OR Exercise, Physical OR Exercises, Physical OR Physical Exercise OR Physical Exercises OR Exercise, Aerobic OR Aerobic Exercise OR Aerobic Exercises OR Exercises, Aerobic OR Exercise, Isometric OR Exercises, Isometric OR Isometric Exercises OR Isometric Exercise OR Acute Exercise OR Acute Exercises OR Exercise, Acute OR Exercises, Acute OR Exercise Training OR Exercise Trainings OR Training, Exercise OR Trainings, Exercise OR Physical Activity OR Activities, Physical OR Activity, Physical OR Physical Activities OR Traditional exercise OR Exercises of Chinese medicine OR TCM exercise OR Tai Ji OR Tai-ji OR Tai Chi OR Chi, Tai OR Tai Ji Quan OR Ji Quan, Tai OR Quan, Tai Ji OR Taiji OR Taijiquan OR T'ai Chi OR Tai Chi Chuan OR Qigong OR Qi Gong OR Ch'i Kung OR Baduanjin OR wuqinxi OR Five-animal play OR Five-animal exercise OR LQG OR Liuzijue OR Six character formula OR Six character code OR Six character pithy OR Yi Jin Jing)
#3	AB,TI=(Randomized Controlled Trial OR Randomised OR Randomized OR Placebo OR RCT OR Random)
#4	#1 AND #2 AND #3

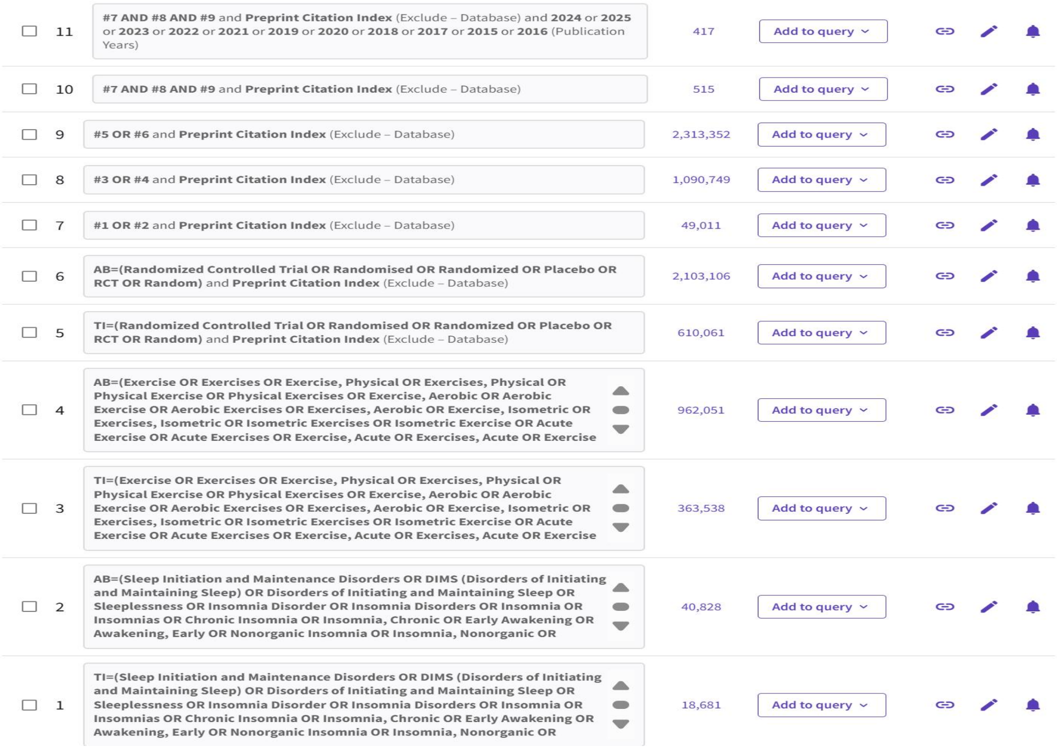

11	#7 AND #8 AND #9 and Preprint Citation Index (Exclude – Database) and 2024 or 2025 or 2023 or 2022 or 2021 or 2019 or 2020 or 2018 or 2017 or 2015 or 2016 (Publication Years)	417	Add to query
10	#7 AND #8 AND #9 and Preprint Citation Index (Exclude – Database)	515	Add to query
9	#5 OR #6 and Preprint Citation Index (Exclude – Database)	2,313,352	Add to query
8	#3 OR #4 and Preprint Citation Index (Exclude – Database)	1,090,749	Add to query
7	#1 OR #2 and Preprint Citation Index (Exclude – Database)	49,011	Add to query
6	AB=(Randomized Controlled Trial OR Randomised OR Randomized OR Placebo OR RCT OR Random) and Preprint Citation Index (Exclude – Database)	2,103,106	Add to query
5	TI=(Randomized Controlled Trial OR Randomised OR Randomized OR Placebo OR RCT OR Random) and Preprint Citation Index (Exclude – Database)	610,061	Add to query
4	AB=(Exercise OR Exercises OR Exercise, Physical OR Exercises, Physical OR Physical Exercise OR Physical Exercises OR Exercise, Aerobic OR Aerobic Exercise OR Aerobic Exercises OR Exercises, Aerobic OR Exercise, Isometric OR Exercises, Isometric OR Isometric Exercises OR Isometric Exercise OR Acute Exercise OR Acute Exercises OR Exercise, Acute OR Exercises, Acute OR Exercise	962,051	Add to query
3	TI=(Exercise OR Exercises OR Exercise, Physical OR Exercises, Physical OR Physical Exercise OR Physical Exercises OR Exercise, Aerobic OR Aerobic Exercise OR Aerobic Exercises OR Exercises, Aerobic OR Exercise, Isometric OR Exercises, Isometric OR Isometric Exercises OR Isometric Exercise OR Acute Exercise OR Acute Exercises OR Exercise, Acute OR Exercises, Acute OR Exercise	363,538	Add to query
2	AB=(Sleep Initiation and Maintenance Disorders OR DIMS (Disorders of Initiating and Maintaining Sleep) OR Disorders of Initiating and Maintaining Sleep OR Sleeplessness OR Insomnia Disorder OR Insomnia Disorders OR Insomnia OR Insomnias OR Chronic Insomnia OR Insomnia, Chronic OR Early Awakening OR Awakening, Early OR Nonorganic Insomnia OR Insomnia, Nonorganic OR	40,828	Add to query
1	TI=(Sleep Initiation and Maintenance Disorders OR DIMS (Disorders of Initiating and Maintaining Sleep) OR Disorders of Initiating and Maintaining Sleep OR Sleeplessness OR Insomnia Disorder OR Insomnia Disorders OR Insomnia OR Insomnias OR Chronic Insomnia OR Insomnia, Chronic OR Early Awakening OR Awakening, Early OR Nonorganic Insomnia OR Insomnia, Nonorganic OR	18,681	Add to query

3. Embase-751

#1	'Exercise':ab,ti OR 'Exercises':ab,ti OR 'Exercise, Physical':ab,ti OR 'Exercises, Physical':ab,ti OR 'Physical Exercise':ab,ti OR 'Physical Exercises':ab,ti OR 'Exercise, Aerobic':ab,ti OR 'Aerobic Exercise':ab,ti OR 'Aerobic Exercises':ab,ti OR 'Exercises, Aerobic':ab,ti OR 'Exercise, Isometric':ab,ti OR 'Exercises, Isometric':ab,ti OR 'Isometric Exercises':ab,ti OR 'Isometric Exercise':ab,ti OR 'Acute Exercise':ab,ti OR 'Acute Exercises':ab,ti OR 'Exercise, Acute':ab,ti OR 'Exercises, Acute':ab,ti OR 'Exercise Training':ab,ti OR 'Exercise Trainings':ab,ti OR 'Training, Exercise':ab,ti OR 'Trainings, Exercise':ab,ti OR 'Physical Activity':ab,ti OR 'Activities, Physical':ab,ti OR 'Activity, Physical':ab,ti OR 'Physical Activities':ab,ti OR 'Traditional exercise':ab,ti OR 'Exercises of Chinese medicine':ab,ti OR 'TCM exercise':ab,ti OR 'Tai Ji':ab,ti OR 'Tai-ji':ab,ti OR 'Tai Chi':ab,ti OR 'Chi, Tai':ab,ti OR 'Tai Ji Quan':ab,ti OR 'Ji Quan, Tai':ab,ti OR 'Quan, Tai Ji':ab,ti OR 'Taiji':ab,ti OR 'Taijiquan':ab,ti OR 'T'ai Chi':ab,ti OR 'Tai Chi Chuan':ab,ti OR 'Qigong':ab,ti OR 'Qi Gong':ab,ti OR 'Ch'i Kung':ab,ti OR 'Baduanjin':ab,ti OR 'wuqinxi':ab,ti OR 'Five-animal play':ab,ti OR 'Five-animal exercise':ab,ti OR 'LQG':ab,ti OR 'Liuzijue':ab,ti OR 'Six character formula':ab,ti OR 'Six character code':ab,ti OR 'Six character pithy':ab,ti OR 'Yi Jin Jing':ab,ti
#2	'Sleep Initiation and Maintenance Disorders':ab,ti OR 'DIMS (Disorders of Initiating and Maintaining Sleep)':ab,ti OR 'Disorders of Initiating and Maintaining Sleep':ab,ti OR 'Sleeplessness':ab,ti OR 'Insomnia Disorder':ab,ti OR 'Insomnia Disorders':ab,ti OR 'Insomnia':ab,ti OR 'Insomnias':ab,ti OR 'Chronic Insomnia':ab,ti OR 'Insomnia, Chronic':ab,ti OR 'Early Awakening':ab,ti OR 'Awakening, Early':ab,ti OR 'Nonorganic Insomnia':ab,ti OR 'Insomnia, Nonorganic':ab,ti OR 'Primary Insomnia':ab,ti OR 'Insomnia, Primary':ab,ti OR 'Psychophysiological Insomnia':ab,ti OR 'Insomnia, Psychophysiological':ab,ti OR 'Rebound Insomnia':ab,ti OR 'Insomnia, Rebound':ab,ti OR 'Secondary Insomnia':ab,ti OR 'Insomnia, Secondary':ab,ti OR 'Sleep Initiation Dysfunction':ab,ti OR 'Dysfunction, Sleep Initiation':ab,ti OR 'Dysfunctions, Sleep Initiation':ab,ti OR 'Sleep Initiation Dysfunctions':ab,ti OR 'Transient Insomnia':ab,ti OR 'Insomnia, Transient':ab,ti
#3	'Randomized Controlled Trial':ab,ti OR 'Randomised':ab,ti OR 'Randomized':ab,ti OR 'Placebo':ab,ti OR 'RCT':ab,ti OR 'Random':ab,ti
#4	#1 AND #2 AND #3

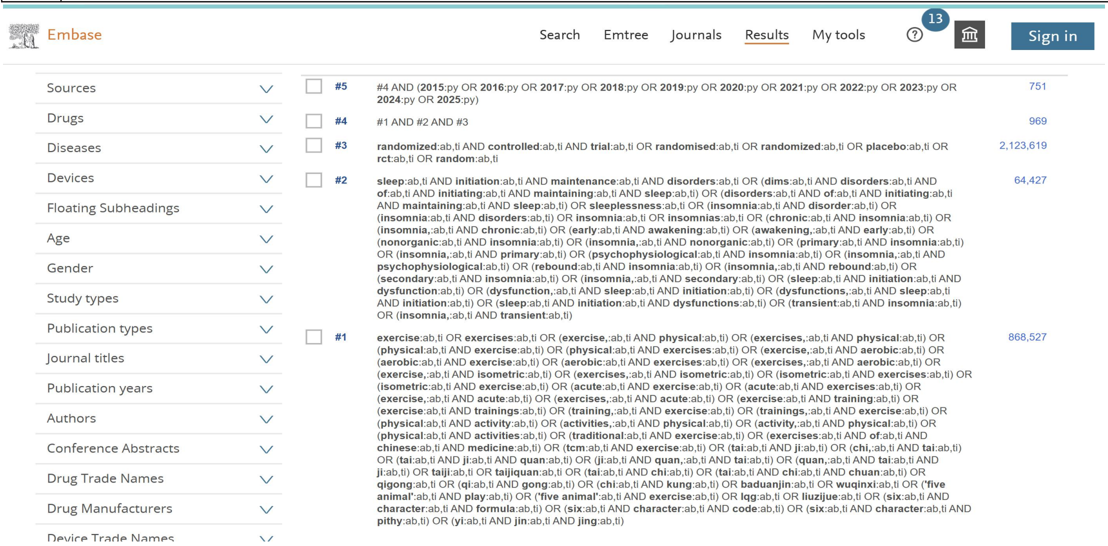

4. ProQuest-245

#1	AB,TI("Exercise" OR "Exercises" OR "Exercise, Physical" OR "Exercises, Physical" OR "Physical Exercise" OR "Physical Exercises" OR "Exercise, Aerobic" OR "Aerobic Exercise" OR "Aerobic Exercises" OR "Exercises, Aerobic" OR "Exercise, Isometric" OR "Exercises, Isometric" OR "Isometric Exercises" OR "Isometric Exercise" OR "Acute Exercise" OR "Acute Exercises" OR "Exercise, Acute" OR "Exercises, Acute" OR "Exercise Training" OR "Exercise Trainings" OR "Training, Exercise" OR "Trainings, Exercise" OR "Physical Activity" OR "Activities, Physical" OR "Activity, Physical" OR "Physical Activities" OR "Traditional exercise" OR "Exercises of Chinese medicine" OR "TCM exercise" OR "Tai Ji" OR "Tai-ji" OR "Tai Chi" OR "Chi, Tai" OR "Tai Ji Quan" OR "Ji Quan, Tai" OR "Quan, Tai Ji" OR "Taiji" OR "Taijiquan" OR "T'ai Chi" OR "Tai Chi Chuan" OR "Qigong" OR "Qi Gong" OR "Ch'i Kung" OR "Baduanjin" OR "wuqinxi" OR "Five-animal play" OR "Five-animal exercise" OR "LQG" OR "Liuzijue" OR "Six character formula" OR "Six character code" OR "Six character pithy" OR "Yi Jin Jing")
#2	AB,TI("Sleep Initiation and Maintenance Disorders" OR "DIMS (Disorders of Initiating and Maintaining Sleep)" OR "Disorders of Initiating and Maintaining Sleep" OR "Sleeplessness" OR "Insomnia Disorder" OR "Insomnia Disorders" OR "Insomnia" OR "Insomnias" OR "Chronic Insomnia" OR "Insomnia, Chronic" OR "Early Awakening" OR "Awakening, Early" OR "Nonorganic Insomnia" OR "Insomnia, Nonorganic" OR "Primary Insomnia" OR "Insomnia, Primary" OR "Psychophysiological Insomnia" OR "Insomnia, Psychophysiological" OR "Rebound Insomnia" OR "Insomnia, Rebound" OR "Secondary Insomnia" OR "Insomnia, Secondary" OR "Sleep Initiation Dysfunction" OR "Dysfunction, Sleep Initiation" OR "Dysfunctions, Sleep Initiation" OR "Sleep Initiation Dysfunctions" OR "Transient Insomnia" OR "Insomnia, Transient")
#3	AB,TI("Randomized Controlled Trial" OR "Randomised" OR "Randomized" OR "Placebo" OR "RCT" OR "Random")
#4	#1 AND #2 AND #3

	集 ▾	检索	数据库	结果	保存检索/提醒	其他操作
☐	S5	⊞ [S2] AND [S3] AND [S4]	18 个数据库	245°	保存检索/提醒 ▾	其他操作 ▾
☐	S4	⊞ AB,TI("Randomized Controlled Trial" OR "Randomised" OR "Randomized" OR "Placebo" OR "RCT" OR "Random") ✔ 应用限制	18 个数据库	657,476*	保存检索/提醒 ▾	其他操作 ▾
☐	S3	⊞ AB,TI("Sleep Initiation and Maintenance Disorders" OR "DIMS (Disorders of Initiating and Maintaining Sleep)" OR "Disorders of Initiating and Maintaining Sleep" OR "Sleeplessness" OR "Insomnia Disorder" OR "Insomnia Disorders" OR "Insomnia" OR "Insomnias" OR "Chronic Insomnia" OR "Insomnia, Chronic" OR "Early Awakening" OR "Awakening, Early" OR "Nonorganic Insomnia" OR "Insomnia, Nonorganic" OR "Primary Insomnia" OR "Insomnia, Primary" OR "Psychophysiological Insomnia" OR "Insomnia, Psychophysiological" OR "Rebound Insomnia" OR "Insomnia, Rebound" OR "Secondary Insomnia" OR "Insomnia, Secondary" OR "Sleep Initiation Dysfunction" OR "Dysfunction, Sleep Initiation" OR "Dysfunctions, Sleep Initiation" OR "Sleep Initiation Dysfunctions" OR "Transient Insomnia" OR "Insomnia, Transient") ✔ 应用限制	18 个数据库	22,119*	保存检索/提醒 ▾	其他操作 ▾
☐	S2	⊞ AB,TI("Exercise" OR "Exercises" OR "Exercise, Physical" OR "Exercises, Physical" OR "Physical Exercise" OR "Physical Exercises" OR "Exercise, Aerobic" OR "Aerobic Exercise" OR "Aerobic Exercises" OR "Exercises, Aerobic" OR "Exercise, Isometric" OR "Exercises, Isometric" OR "Isometric Exercises" OR "Isometric Exercise" OR "Acute Exercise" OR "Acute Exercises" OR "Exercise, Acute" OR "Exercises, Acute" OR "Exercise Training" OR "Exercise Trainings" OR "Training, Exercise" OR "Trainings, Exercise" OR "Physical Activity" OR "Activities, Physical" OR "Activity, Physical" OR "Physical Activities" OR "Traditional exercise" OR "Exercises of Chinese medicine" OR "TCM exercise" OR "Tai Ji" OR "Tai-ji" OR "Tai Chi" OR "Chi, Tai" OR "Tai Ji Quan" OR "Ji Quan, Tai" OR "Quan, Tai Ji" OR "Taiji" OR "Taijiquan" OR "T'ai Chi" OR "Tai Chi Chuan" OR "Qigong" OR "Qi Gong" OR "Ch'i Kung" OR "Baduanjin" OR "wuqinxi" OR "Five-animal play" OR "Five-animal exercise" OR "LQG" OR "Liuzijue" OR "Six character formula" OR "Six character code" OR "Six character pithy" OR "Yi Jin Jing") ✔ 应用限制	18 个数据库	314,957*	保存检索/提醒 ▾	其他操作 ▾

5. Ovid-130

#1	"Exercise" OR "Exercises" OR "Exercise, Physical" OR "Exercises, Physical" OR "Physical Exercise" OR "Physical Exercises" OR "Exercise, Aerobic" OR "Aerobic Exercise" OR "Aerobic Exercises" OR "Exercises, Aerobic" OR "Exercise, Isometric" OR "Exercises, Isometric" OR "Isometric Exercises" OR "Isometric Exercise" OR "Acute Exercise" OR "Acute Exercises" OR "Exercise, Acute" OR "Exercises, Acute" OR "Exercise Training" OR "Exercise Trainings" OR "Training, Exercise" OR "Trainings, Exercise" OR "Physical Activity" OR "Activities, Physical" OR "Activity, Physical" OR "Physical Activities" OR "Traditional exercise" OR "Exercises of Chinese medicine" OR "TCM exercise" OR "Tai Ji" OR "Tai-ji" OR "Tai Chi" OR "Chi, Tai" OR "Tai Ji Quan" OR "Ji Quan, Tai" OR "Quan, Tai Ji" OR "Taiji" OR "Taijiquan" OR "T'ai Chi" OR "Tai Chi Chuan" OR "Qigong" OR "Qi Gong" OR "Ch'i Kung" OR "Baduanjin" OR "wuqinxi" OR "Five-animal play" OR "Five-animal exercise" OR "LQG" OR "Liuzijue" OR "Six character formula" OR "Six character code" OR "Six character pithy" OR "Yi Jin Jing"
#2	"Sleep Initiation and Maintenance Disorders" OR "DIMS (Disorders of Initiating and Maintaining Sleep)" OR "Disorders of Initiating and Maintaining Sleep" OR "Sleeplessness" OR "Insomnia Disorder" OR "Insomnia Disorders" OR "Insomnia" OR "Insomnias" OR "Chronic Insomnia" OR "Insomnia, Chronic" OR "Early Awakening" OR "Awakening, Early" OR "Nonorganic Insomnia" OR "Insomnia, Nonorganic" OR "Primary Insomnia" OR "Insomnia, Primary" OR "Psychophysiological Insomnia" OR "Insomnia, Psychophysiological" OR "Rebound Insomnia" OR "Insomnia, Rebound" OR "Secondary Insomnia" OR "Insomnia, Secondary" OR "Sleep Initiation Dysfunction" OR "Dysfunction, Sleep Initiation" OR "Dysfunctions, Sleep Initiation" OR "Sleep Initiation Dysfunctions" OR "Transient Insomnia" OR "Insomnia, Transient"
#3	"Randomized Controlled Trial" OR "Randomised" OR "Randomized" OR "Placebo" OR "RCT" OR "Random"
#4	#1 AND #2 AND #3

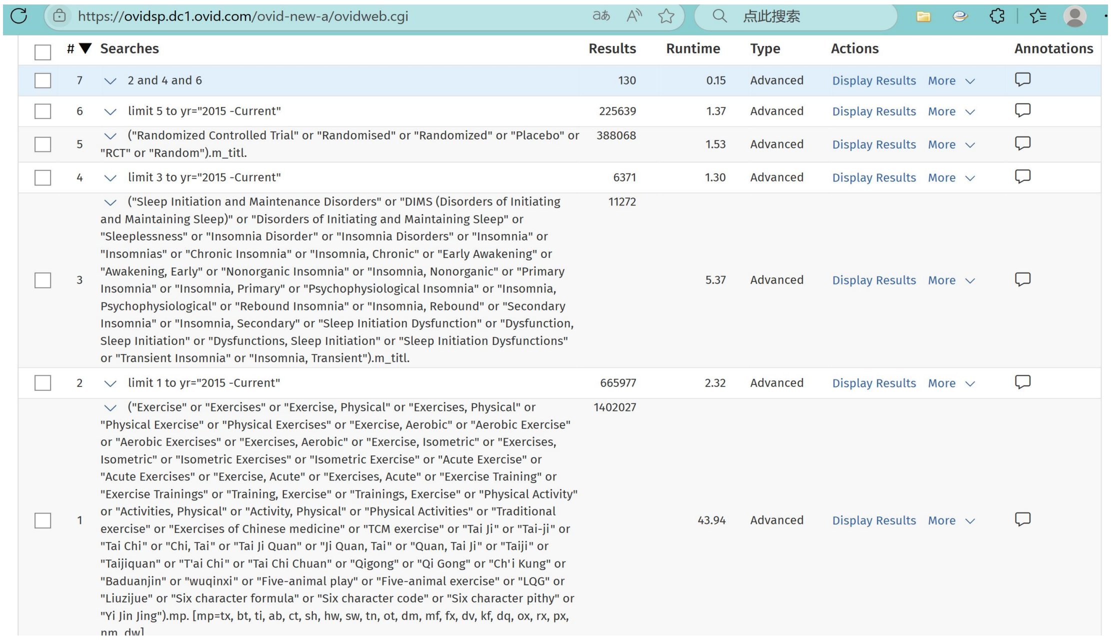
https://ovidsp.dc1.ovid.com/ovid-new-a/ovidweb.cgi

#	Searches	Results	Runtime	Type	Actions	Annotations
7	2 and 4 and 6	130	0.15	Advanced	Display Results More	
6	limit 5 to yr="2015 -Current"	225639	1.37	Advanced	Display Results More	
5	("Randomized Controlled Trial" or "Randomised" or "Randomized" or "Placebo" or "RCT" or "Random").m_titl.	388068	1.53	Advanced	Display Results More	
4	limit 3 to yr="2015 -Current"	6371	1.30	Advanced	Display Results More	
3	("Sleep Initiation and Maintenance Disorders" or "DIMS (Disorders of Initiating and Maintaining Sleep)" or "Disorders of Initiating and Maintaining Sleep" or "Sleeplessness" or "Insomnia Disorder" or "Insomnia Disorders" or "Insomnia" or "Insomnias" or "Chronic Insomnia" or "Insomnia, Chronic" or "Early Awakening" or "Awakening, Early" or "Nonorganic Insomnia" or "Insomnia, Nonorganic" or "Primary Insomnia" or "Insomnia, Primary" or "Psychophysiological Insomnia" or "Insomnia, Psychophysiological" or "Rebound Insomnia" or "Insomnia, Rebound" or "Secondary Insomnia" or "Insomnia, Secondary" or "Sleep Initiation Dysfunction" or "Dysfunction, Sleep Initiation" or "Dysfunctions, Sleep Initiation" or "Sleep Initiation Dysfunctions" or "Transient Insomnia" or "Insomnia, Transient").m_titl.	11272	5.37	Advanced	Display Results More	
2	limit 1 to yr="2015 -Current"	665977	2.32	Advanced	Display Results More	
1	("Exercise" or "Exercises" or "Exercise, Physical" or "Exercises, Physical" or "Physical Exercise" or "Physical Exercises" or "Exercise, Aerobic" or "Aerobic Exercise" or "Aerobic Exercises" or "Exercises, Aerobic" or "Exercise, Isometric" or "Exercises, Isometric" or "Isometric Exercises" or "Isometric Exercise" or "Acute Exercise" or "Acute Exercises" or "Exercise, Acute" or "Exercises, Acute" or "Exercise Training" or "Exercise Trainings" or "Training, Exercise" or "Trainings, Exercise" or "Physical Activity" or "Activities, Physical" or "Activity, Physical" or "Physical Activities" or "Traditional exercise" or "Exercises of Chinese medicine" or "TCM exercise" or "Tai Ji" or "Tai-ji" or "Tai Chi" or "Chi, Tai" or "Tai Ji Quan" or "Ji Quan, Tai" or "Quan, Tai Ji" or "Taiji" or "Taijiquan" or "T'ai Chi" or "Tai Chi Chuan" or "Qigong" or "Qi Gong" or "Ch'i Kung" or "Baduanjin" or "wuqinxi" or "Five-animal play" or "Five-animal exercise" or "LQG" or "Liuzijue" or "Six character formula" or "Six character code" or "Six character pithy" or "Yi Jin Jing").mp. [mp=tx, bt, ti, ab, ct, sh, hw, sw, tn, ot, dm, mf, fx, dv, kf, dq, ox, rx, px, nm, dw]	1402027	43.94	Advanced	Display Results More	

6. Scopus-1011

#1	"Exercise" OR "Exercises" OR "Exercise, Physical" OR "Exercises, Physical" OR "Physical Exercise" OR "Physical Exercises" OR "Exercise, Aerobic" OR "Aerobic Exercise" OR "Aerobic Exercises" OR "Exercises, Aerobic" OR "Exercise, Isometric" OR "Exercises, Isometric" OR "Isometric Exercises" OR "Isometric Exercise" OR "Acute Exercise" OR "Acute Exercises" OR "Exercise, Acute" OR "Exercises, Acute" OR "Exercise Training" OR "Exercise Trainings" OR "Training, Exercise" OR "Trainings, Exercise" OR "Physical Activity" OR "Activities, Physical" OR "Activity, Physical" OR "Physical Activities" OR "Traditional exercise" OR "Exercises of Chinese medicine" OR "TCM exercise" OR "Tai Ji" OR "Tai-ji" OR "Tai Chi" OR "Chi, Tai" OR "Tai Ji Quan" OR "Ji Quan, Tai" OR "Quan, Tai Ji" OR "Taiji" OR "Taijiquan" OR "T'ai Chi" OR "Tai Chi Chuan" OR "Qigong" OR "Qi Gong" OR "Ch'i Kung" OR "Baduanjin" OR "wuqinxi" OR "Five-animal play" OR "Five-animal exercise" OR "LQG" OR "Liuzijue" OR "Six character formula" OR "Six character code" OR "Six character pithy" OR "Yi Jin Jing"
#2	"Sleep Initiation and Maintenance Disorders" OR "DIMS (Disorders of Initiating and Maintaining Sleep)" OR "Disorders of Initiating and Maintaining Sleep" OR "Sleeplessness" OR "Insomnia Disorder" OR "Insomnia Disorders" OR "Insomnia" OR "Insomnias" OR "Chronic Insomnia" OR "Insomnia, Chronic" OR "Early Awakening" OR "Awakening, Early" OR "Nonorganic Insomnia" OR "Insomnia, Nonorganic" OR "Primary Insomnia" OR "Insomnia, Primary" OR "Psychophysiological Insomnia" OR "Insomnia, Psychophysiological" OR "Rebound Insomnia" OR "Insomnia, Rebound" OR "Secondary Insomnia" OR "Insomnia, Secondary" OR "Sleep Initiation Dysfunction" OR "Dysfunction, Sleep Initiation" OR "Dysfunctions, Sleep Initiation" OR "Sleep Initiation Dysfunctions" OR "Transient Insomnia" OR "Insomnia, Transient"
#3	"Randomized Controlled Trial" OR "Randomised" OR "Randomized" OR "Placebo" OR "RCT" OR "Random"
#4	#1 AND #2 AND #3

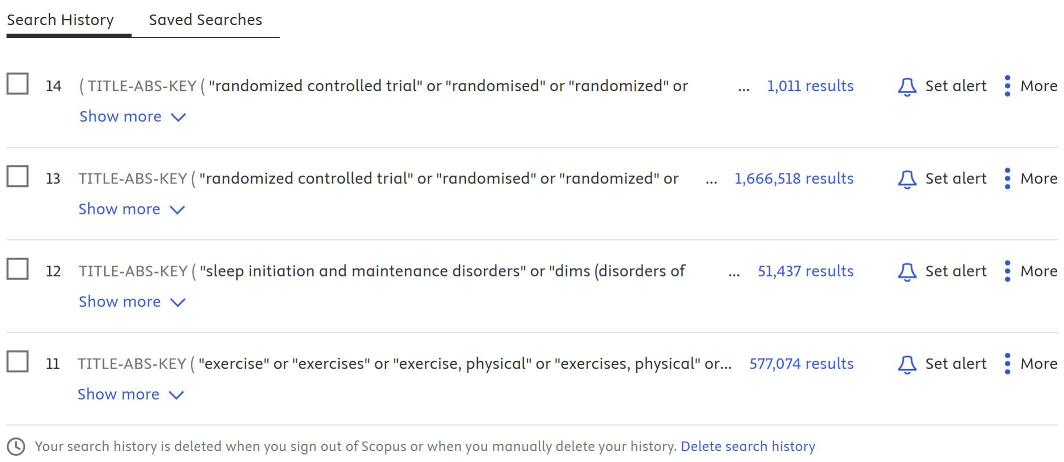

7. CINAHL-94

#1	XB("Exercise" OR "Exercises" OR "Exercise, Physical" OR "Exercises, Physical" OR "Physical Exercise" OR "Physical Exercises" OR "Exercise, Aerobic" OR "Aerobic Exercise" OR "Aerobic Exercises" OR "Exercises, Aerobic" OR "Exercise, Isometric" OR "Exercises, Isometric" OR "Isometric Exercises" OR "Isometric Exercise" OR "Acute Exercise" OR "Acute Exercises" OR "Exercise, Acute" OR "Exercises, Acute" OR "Exercise Training" OR "Exercise Trainings" OR "Training, Exercise" OR "Trainings, Exercise" OR "Physical Activity" OR "Activities, Physical" OR "Activity, Physical" OR "Physical Activities" OR "Traditional exercise" OR "Exercises of Chinese medicine" OR "TCM exercise" OR "Tai Ji" OR "Tai-ji" OR "Tai Chi" OR "Chi, Tai" OR "Tai Ji Quan" OR "Ji Quan, Tai" OR "Quan, Tai Ji" OR "Taiji" OR "Taijiquan" OR "T'ai Chi" OR "Tai Chi Chuan" OR "Qigong" OR "Qi Gong" OR "Ch'i Kung" OR "Baduanjin" OR "wuqinxi" OR "Five-animal play" OR "Five-animal exercise" OR "LQG" OR "Liuzijue" OR "Six character formula" OR "Six character code" OR "Six character pithy" OR "Yi Jin Jing")
#2	XB("Sleep Initiation and Maintenance Disorders" OR "DIMS (Disorders of Initiating and Maintaining Sleep)" OR "Disorders of Initiating and Maintaining Sleep" OR "Sleeplessness" OR "Insomnia Disorder" OR "Insomnia Disorders" OR "Insomnia" OR "Insomnias" OR "Chronic Insomnia" OR "Insomnia, Chronic" OR "Early Awakening" OR "Awakening, Early" OR "Nonorganic Insomnia" OR "Insomnia, Nonorganic" OR "Primary Insomnia" OR "Insomnia, Primary" OR "Psychophysiological Insomnia" OR "Insomnia, Psychophysiological" OR "Rebound Insomnia" OR "Insomnia, Rebound" OR "Secondary Insomnia" OR "Insomnia, Secondary" OR "Sleep Initiation Dysfunction" OR "Dysfunction, Sleep Initiation" OR "Dysfunctions, Sleep Initiation" OR "Sleep Initiation Dysfunctions" OR "Transient Insomnia" OR "Insomnia, Transient")
#3	XB("Randomized Controlled Trial" OR "Randomised" OR "Randomized" OR "Placebo" OR "RCT" OR "Random")
#4	#1 AND #2 AND #3

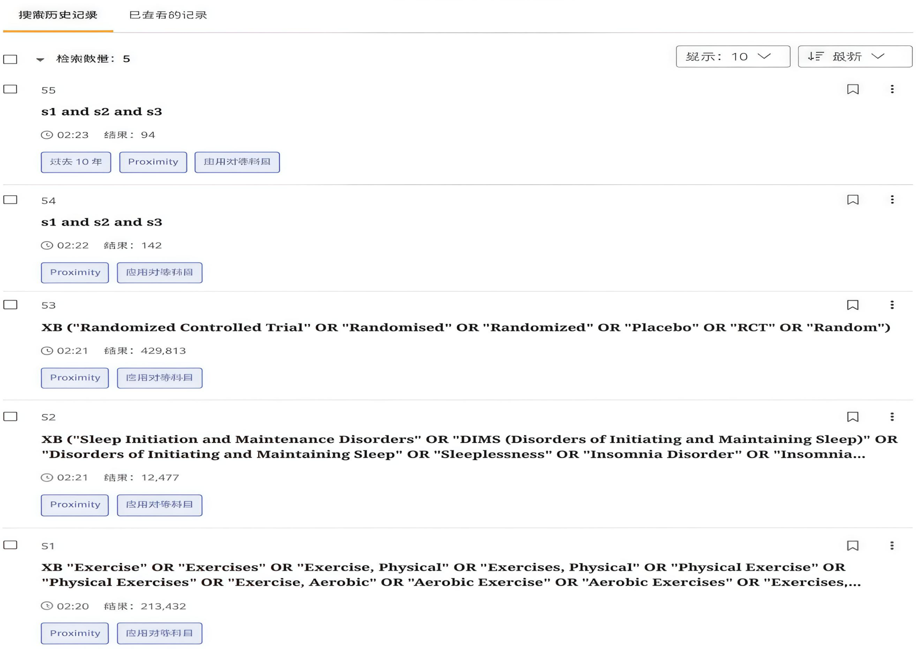

8. Pubmed-309

#1	Sleep Initiation and Maintenance Disorders OR DIMS (Disorders of Initiating and Maintaining Sleep) OR Disorders of Initiating and Maintaining Sleep OR Sleeplessness OR Insomnia Disorder OR Insomnia Disorders OR Insomnia OR Insomnias OR Chronic Insomnia OR Insomnia, Chronic OR Early Awakening OR Awakening, Early OR Nonorganic Insomnia OR Insomnia, Nonorganic OR Primary Insomnia OR Insomnia, Primary OR Psychophysiological Insomnia OR Insomnia, Psychophysiological OR Rebound Insomnia OR Insomnia, Rebound OR Secondary Insomnia OR Insomnia, Secondary OR Sleep Initiation Dysfunction OR Dysfunction, Sleep Initiation OR Dysfunctions, Sleep Initiation OR Sleep Initiation Dysfunctions OR Transient Insomnia OR Insomnia, Transient
#2	Exercise OR Exercises OR Exercise, Physical OR Exercises, Physical OR Physical Exercise OR Physical Exercises OR Exercise, Aerobic OR Aerobic Exercise OR Aerobic Exercises OR Exercises, Aerobic OR Exercise, Isometric OR Exercises, Isometric OR Isometric Exercises OR Isometric Exercise OR Acute Exercise OR Acute Exercises OR Exercise, Acute OR Exercises, Acute OR Exercise Training OR Exercise Trainings OR Training, Exercise OR Trainings, Exercise OR Physical Activity OR Activities, Physical OR Activity, Physical OR Physical Activities OR Traditional exercise OR Exercises of Chinese medicine OR TCM exercise OR Tai Ji OR Tai-ji OR Tai Chi OR Chi, Tai OR Tai Ji Quan OR Ji Quan, Tai OR Quan, Tai Ji OR Taiji OR Taijiquan OR T'ai Chi OR Tai Chi Chuan OR Qigong OR Qi Gong OR Ch'i Kung OR Baduanjin OR wuqinxi OR Five-animal play OR Five-animal exercise OR LQG OR Liuzijue OR Six character formula OR Six character code OR Six character pithy OR Yi Jin Jing
#3	Randomized Controlled Trial OR Randomised OR Randomized OR Placebo OR RCT OR Random
#4	#1 AND #2 AND #3

History and Search Details Download Delete

Search	Actions	Details	Query	Results	Time
#4	···	>	Search: **#1 AND #2 AND #3** Fiiters: **in the last 10 years**	309	23:35:11
#3	···	>	Search: **Randomized Controlled Trial[Title/Abstract] OR Randomised[Title/Abstract] OR Randomized[Title/Abstract] OR Placebo[Title/Abstract] OR RCT[Title/Abstract] OR Random[Title/Abstract]** Fiiters: **in the last 10 years**	723,978	23:34:21
#2	···	>	Search: **Exercise[Title/Abstract] OR Exercises[Title/Abstract] OR Exercise, Physical[Title/Abstract] OR Exercises, Physical[Title/Abstract] OR Physical Exercise[Title/Abstract] OR Physical Exercises[Title/Abstract] OR Exercise, Aerobic[Title/Abstract] OR Aerobic Exercise[Title/Abstract] OR Aerobic Exercises[Title/Abstract] OR Exercises, Aerobic[Title/Abstract] OR Exercise, Isometric[Title/Abstract] OR Exercises, Isometric[Title/Abstract] OR Isometric Exercises[Title/Abstract] OR Isometric Exercise[Title/Abstract] OR Acute Exercise[Title/Abstract] OR Acute Exercises[Title/Abstract] OR Exercise, Acute[Title/Abstract] OR Exercises, Acute[Title/Abstract] OR Exercise Training[Title/Abstract] OR Exercise Trainings[Title/Abstract] OR Training, Exercise[Title/Abstract] OR Trainings, Exercise[Title/Abstract] OR Physical Activity[Title/Abstract] OR Activities, Physical[Title/Abstract] OR Activity, Physical[Title/Abstract] OR Physical Activities[Title/Abstract] OR Traditional exercise[Title/Abstract] OR Exercises of Chinese medicine[Title/Abstract] OR TCM exercise[Title/Abstract] OR Tai Ji[Title/Abstract] OR Tai-ji[Title/Abstract] OR Tai Chi[Title/Abstract] OR Chi, Tai[Title/Abstract] OR Tai Ji Quan[Title/Abstract] OR Ji Quan, Tai[Title/Abstract] OR Quan, Tai Ji[Title/Abstract] OR Taiji[Title/Abstract] OR Taijiquan[Title/Abstract] OR T'ai Chi[Title/Abstract] OR Tai Chi Chuan[Title/Abstract] OR Qigong[Title/Abstract] OR Qi Gong[Title/Abstract] OR Ch'i Kung[Title/Abstract] OR Baduanjin[Title/Abstract] OR wuqinxi[Title/Abstract] OR Five-animal play[Title/Abstract] OR Five-animal exercise[Title/Abstract] OR LQG[Title/Abstract] OR Liuzijue[Title/Abstract] OR Six character formula[Title/Abstract] OR Six character code[Title/Abstract] OR Six character pithy[Title/Abstract] OR Yi Jin Jing[Title/Abstract]** Filters: **in the last 10 years**	269,974	23:23:38
#1	···	>	Search: **Sleep Initiation[Title/Abstract] AND Maintenance Disorders[Title/Abstract] OR DIMS (Disorders of Initiating[Title/Abstract] AND Maintaining Sleep[Title/Abstract]) OR Disorders of Initiating[Title/Abstract] AND Maintaining Sleep[Title/Abstract] OR Sleeplessness[Title/Abstract] OR Insomnia Disorder[Title/Abstract] OR Insomnia Disorders[Title/Abstract] OR Insomnia[Title/Abstract] OR Insomnias[Title/Abstract] OR Chronic Insomnia[Title/Abstract] OR Insomnia, Chronic[Title/Abstract] OR Early Awakening[Title/Abstract] OR Awakening, Early[Title/Abstract] OR Nonorganic Insomnia[Title/Abstract] OR Insomnia, Nonorganic[Title/Abstract] OR Primary Insomnia[Title/Abstract] OR Insomnia, Primary[Title/Abstract] OR Psychophysiological Insomnia[Title/Abstract] OR Insomnia, Psychophysiological[Title/Abstract] OR Rebound Insomnia[Title/Abstract] OR Insomnia, Rebound[Title/Abstract] OR Secondary Insomnia[Title/Abstract] OR Insomnia, Secondary[Title/Abstract] OR Sleep Initiation Dysfunction[Title/Abstract] OR Dysfunction, Sleep Initiation[Title/Abstract] OR Dysfunctions, Sleep Initiation[Title/Abstract] OR Sleep Initiation Dysfunctions[Title/Abstract] OR Transient Insomnia[Title/Abstract] OR Insomnia, Transient[Title/Abstract]** Fiiters: **in the last 10 years**	22,404	23:23:15

Showing 1 to 4 of 4 entries
