# Supplementary material for: Physical activity for insomnia: a scoping review within the Nursing Science Precision Health model
Source: Front Public Health. 2026 May 28;14:1834146. doi: 10.3389/fpubh.2026.1834146 (PMC13253286; doi:10.3389/fpubh.2026.1834146)
Supplement: Supplementary file 3 [file Supplementary_File_3.pdf]

Appendix C: Overview of Cochrane Risk of Bias Assessment for Included Studies

Study	Selection bias	Selection bias	Performance bias	Detection bias	Attrition bias	Reporting bias	Other bias
Rozales et al. 2024 [26]	Low risk	Unclear risk	Unclear risk	Unclear risk	Low risk	Low risk	Low risk
Cammalleri, et al. 2024 [27]	Low risk	Low risk	High risk	Unclear risk	Low risk	Low risk	Low risk
Baron et al. 2023 [28]	Low risk	Low risk	High risk	Unclear risk	Low risk	Low risk	Low risk
Chin et al. 2022 [29]	Low risk	Low risk	Unclear risk	Low risk	Low risk	Low risk	Low risk
Ferreira et al. 2022 [30]	Low risk	Unclear risk	Unclear risk	Unclear risk	Low risk	Low risk	Low risk
Tseng et al. 2020 [31]	Low risk	Low risk	Unclear risk	Low risk	Low risk	Unclear risk	Low risk
Abd et al.2020 [32]	Unclear risk	Unclear risk	Unclear risk	Unclear risk	Low risk	Unclear risk	Low risk
Niu et al.2020 [33]	Low risk	Low risk	Unclear risk	Low risk	Low risk	Low risk	Low risk
Eshaghi et al.2020 [34]	Unclear risk	Unclear risk	Unclear risk	Unclear risk	Unclear risk	Unclear risk	Low risk
Jamshidi et al.2019[35]	Unclear risk	Unclear risk	Unclear risk	Unclear risk	Unclear risk	Unclear risk	Low risk
Chen et al. 2019 [36]	Low risk	Low risk	High risk	Unclear risk	Low risk	Low risk	Low risk
El-Kader et al. 2019 [37]	Unclear risk	Unclear risk	Unclear risk	Unclear risk	Unclear risk	Unclear risk	Low risk
Iuliana et al. 2019 [38]	Low risk	Unclear risk	Unclear risk	Unclear risk	Low risk	Unclear risk	Low risk
Taheri et al. 2018 [39]	Unclear risk	Unclear risk	Unclear risk	Unclear risk	Unclear risk	Unclear risk	Low risk
Li-Jung Chen 2016 [40]	Unclear risk	Unclear risk	Unclear risk	Unclear risk	High risk	Unclear risk	Low risk
Saba et al. 2016 [41]	Low risk	Unclear risk	Unclear risk	Unclear risk	Low risk	Unclear risk	Low risk
Tan et al. 2016 [42]	Low risk	Unclear risk	Unclear risk	Unclear risk	Low risk	Low risk	Low risk
IULIANA et al. 2015 [43]	Low risk	Low risk	Unclear risk	Unclear risk	Low risk	Unclear risk	Low risk
Jihui et al. 2015 [44]	Low risk	Low risk	Unclear risk	Low risk	Low risk	Unclear risk	Low risk
Farkhondeh et al. 2015 [45]	Low risk	Unclear risk	Unclear risk	Unclear risk	Low risk	Unclear risk	Low risk
Camila et al. 2024 [46]	Unclear risk	Low risk	High risk	Unclear risk	Low risk	Low risk	Low risk
Samuel et al. 2017 [47]	Low risk	Low risk	Low risk	Low risk	Low risk	Low risk	Low risk
Jiali et al. 2024 [48]	Low risk	Unclear risk	High risk	Unclear risk	Low risk	Low risk	Low risk
Jiali et al. 2024 [49]	Low risk	Unclear risk	High risk	Low risk	Low risk	Low risk	Low risk
Siu et al. 2021 [50]	Low risk	Low risk	Unclear risk	Unclear risk	Low risk	Low risk	Low risk
Judith et al. 2015 [51]	Low risk	Low risk	High risk	Unclear risk	Low risk	Low risk	Low risk
Kanika et al. 2023 [52]	Low risk	Unclear risk	Unclear risk	Unclear risk	Low risk	Low risk	Low risk
Agustín et al. 2019 [53]	Low risk	Low risk	Unclear risk	Low risk	Low risk	Low risk	Low risk
Wing-Fai et al. 2025 [54]	Low risk	Low risk	High risk	Low risk	Low risk	Low risk	Low risk
Yuan-Gao et al. 2022 [55]	Low risk	Unclear risk	Unclear risk	Unclear risk	Low risk	Low risk	Low risk
Wing-Fai et al. 2018 [56]	Low risk	Low risk	Unclear risk	Low risk	Low risk	Low risk	Low risk
Glauber Sá et al. 2018 [57]	Low risk	Low risk	Unclear risk	Low risk	Low risk	Low risk	Low risk
