# Supplementary material for: Physical activity for insomnia: a scoping review within the Nursing Science Precision Health model
Source: Front Public Health. 2026 May 28;14:1834146. doi: 10.3389/fpubh.2026.1834146 (PMC13253286; doi:10.3389/fpubh.2026.1834146)
Supplement: Supplementary file 4 [file Supplementary_File_4.pdf]

Appendix D: Mapping of included studies across NSPH domains

Study/Year	Symptom	Phenotype	Biomarker	Intervention
Rozales et al. 2024 [26]	PSG, actigraphy, sleep diary	NA	Cortisol	Aerobic exercise
Cammalleri, et al. 2024 [27]	PSG, ISI, PSQI, ESS	NA	NA	Aerobic exercise + resistance training
Baron et al. 2023 [28]	ISI, ESS, Daily sleep diary	Anxiety, depression, stress	Core temperature	Aerobic exercise
Chin et al. 2022 [29]	PSQI, ISI, wGT3X-BT actigraph, sleep diary	Anxiety, depression	NA	Aerobic exercise
Ferreira et al. 2022 [30]	ISI, PSQI, PSG, sleep diary	Anxiety, depression, quality of life	Cortisol	Aerobic exercise
Tseng et al. 2020 [31]	PSQI, actigraph	Depression, cognitive function	HRV	Aerobic exercise
Abd et al.2020 [32]	PSG	Anxiety, depression, self-esteem, mood states	CD3+, CD4+, CD8+ T cells count	Aerobic exercise
Niu et al.2020 [33]	PSQI, 19-item MEQ, actigraphy	NA	NA	Aerobic exercise
Eshaghi et al.2020 [34]	PSQI	Cognitive function	NA	Aerobic exercise
Jamshidi et al.2019[35]	PSQI	NA	NA	Aerobic exercise
Chen et al. 2019 [36]	Heart rate monitor, actigraph	NA	NA	Aerobic exercise
El-Kader et al. 2019 [37]	PSG	NA	IL-6, TNF-α, IL-10	Aerobic exercise
Iuliana et al. 2019 [38]	ISI, ESS, actigraphy	Anxiety, depression, quality of life, fatigue	NA	Aerobic exercise
Taheri et al. 2018 [39]	PSQI	NA	NA	Aerobic exercise
Li-Jung Chen 2016 [40]	Sleep actigraphy	NA	NA	Aerobic/strength exercise
Saba et al. 2016 [41]	PSQI, ESS	NA	NA	Aerobic exercise
Tan et al. 2016 [42]	BNSQ, ESS, sleep diary	NA	NA	Aerobic exercise
IULIANA et al. 2015 [43]	ISI, ESS, actigraphy	Anxiety, depression, quality of life, fatigue	BMI	Aerobic exercise
Jihui et al. 2015 [44]	ISI, ESS, sleep diary	Fatigue	NA	Aerobic exercise
Farkhondeh et al. 2015 [45]	PSQI	Fatigue	NA	Aerobic exercise
Camila et al. 2024 [46]	ISI, PSQI, ESS	Anxiety, depression, mood states	NA	Strength training
Samuel et al. 2017 [47]	ISI, sleep diary	NA	NA	Strength training
Jiali et al. 2024 [48]	ISI, PSAS, EEG, actigraphy, sleep diary	Anxiety, depression, stress, quality of life	NA	Flexibility training
Jiali et al. 2024 [49]	ISI, ESS, PSAS, actigraphy, sleep diary	Anxiety, depression, stress, quality of life	NA	Flexibility training
Siu et al. 2021 [50]	actigraphy, PSQI, ISI, sleep diary	NA	NA	Flexibility training
Judith et al. 2015 [51]	PSQI, sleep diary	NA	Inflammatory marker	Flexibility training
Kanika et al. 2023 [52]	PSQI	Cognitive function, stress, quality of life	NA	Balance and coordination training
Agustín et al. 2019 [53]	PSQI	Anxiety, depression, cognitive function	NA	Balance and coordination training
Wing-Fai et al. 2025 [54]	ISI, actigraphy, sleep diary	Anxiety, depression, fatigue, quality of life	NA	Functional training
Yuan-Gao et al. 2022 [55]	PSQI, ISI, ESS	Anxiety, depression	NA	Functional training
Wing-Fai et al. 2018 [56]	ISI, actigraphy, sleep diary	Anxiety, depression, fatigue, quality of life	NA	Functional training
Glauber Sá et al. 2018 [57]	PSQI, ESS	Cognitive function	NA	Functional training

Abbreviation: ISI: Insomnia Severity Index; PSQI: Pittsburgh Sleep Quality Index; ESS: Epworth Sleepiness Scale; BNSQ: Bergen Insomnia Scale Questionnaire; 19-item MEQ: 19-item Morningness-Eveningness Questionnaire; RBDS: Rapid Eye Movement Sleep Behavior Disorder Scale; PSAS: the Pre-Sleep Arousal Scale; PSG: Polysomnography; EEG: Electroencephalogram; Not Available.
