# Supplementary material for: Physical activity for insomnia: a scoping review within the Nursing Science Precision Health model
Source: Front Public Health. 2026 May 28;14:1834146. doi: 10.3389/fpubh.2026.1834146 (PMC13253286; doi:10.3389/fpubh.2026.1834146)
Supplement: Supplementary file 5 [file Supplementary_File_5.pdf]

Appendix E: Characteristics of Included Physical Activity Interventions

Author/ Year	Country	Sample size	Age	Female %	Sleep Diagnosis	Arms	Exercise Group (F、I、T、T)	Control Group (F、I、T、T)	Intervention Provider / Setting / Supervision
Rozales et al. 2024 [26]	Brazil	17	I:43.3±8.7<br>C:47.3±7.1	NA	DSM-V	2 arms	**Acute aerobic exercise**<br>F: only once<br>I: 50% HRR 5 bpm<br>T: 50 minutes<br>T: treadmill movement	**Acute Zolpidem Intake**<br>10 mg zolpidem at bedtime	• NA<br>• Indoor treadmill<br>• Fully supervised
Cammalleri, et al. 2024 [27]	Canada	19	56±13<br>I:61±10<br>C:50±14	11(57.8)<br>I:6 (60)<br>C:5 (55.5)	DSM-5	2 arms	**Aerobic exercise +resistance training**<br>F: 3x/week<br>I: moderate intensity<br>T: 75 min/session for 16 weeks<br>T: aerobic exercise+6-8 different resistance training	**Relaxation training**<br>F: 3x/week<br>I: NA<br>T: 3x/week for 8 weeks<br>T: diaphragmatic breathing exercises, progressive muscle relaxation, and guided imagery	• Trained exercise physiologist<br>• Home and community<br>• Partially supervised (1 weekly supervised + 2 unsupervised sessions)
Baron et al. 2023 [28]	France	24	I:46.4±5.67<br>C:44.8±7.82	24(100)<br>I:12 (100)<br>C:12(100)	DSM-5	2 arms	**Aerobic exercise**<br>F: 3x/week<br>I: 70 maxHF(50min), >80% maxHF(25min)<br>T: 75 min/session for 12 weeks<br>T: Active walking / treadmill	**Keep up usual lifestyle**	• Experienced trainer<br>• Outdoor/Indoor treadmill<br>• Supervised with individualized HR monitoring and RPE adjustment
Chin et al. 2022 [29]	China (Hong Kong)	75	I_1:65.9±7.0<br>I_2:63.7±4.7<br>I_3:61.5±6.1<br>I_4:61.7±2.7<br>C:63.8±6.0	I_1:7(78)<br>I_2:7(78)<br>I_3:7(70)<br>I_4:9(100)<br>C:8(89)	DSM-5	5 arms	**Aerobic exercise**<br>F: 1-3x/week<br>I: 3.25/6.5METs<br>T: MOD: 150 min/week; VIG: 75 min/week, for 12 weeks<br>T: treadmill	**Stretching exercises**<br>F: 1x/week<br>I: NA<br>T: 75 min/session for 12 weeks<br>T: NA	• Certified coaches/exercise physiologists<br>• Indoor treadmill<br>• Fully supervised (with continuous HR monitoring)
Ferreira et al. 2022 [30]	Brazil	26	I:46.6±8.3<br>C:43.2±10.7	13（81.25)<br>I:6（75)<br>C:7（87.5)	DSM-V<br>ICSD-3	2 arms	**Aerobic exercise**<br>F: 3x/week<br>I: 50% HRR ±5 bpm<br>T: 50 min/session for 12 weeks<br>T: Treadmill	**Aerobic exercise**<br>same as intervention group<br>**Acupuncture**<br>F: 2x/week<br>T: 50 min/session for 12 weeks	• NA<br>• Indoor treadmill<br>• Fully supervised
Tseng et al. 2020 [31]	China (Taiwan)	40	61.7±7.0<br>I:61.1±6.8<br>C:62.2 ±7.4	33(82.5)<br>I:15(75)<br>C:18(90)	PSQI>5	2 arms	**Aerobic exercise**<br>F: 3x/week<br>I: 40%~60% VO2peak<br>T: 50 min/session for 12 weeks<br>T: treadmill walking and stretching exercises	**Keep up usual lifestyle**	• Physical therapist<br>• Laboratory<br>• Fully supervised

Abd et al.2020 [32]	Saudi Arabia.	80	I:51.27±5.32<br>C:52.64±4.81	I:26(65)<br>C:28(70)	Chronic primary insomnia ⩾ six months	2 arms	**Aerobic exercise**<br>F: 3x/week<br>I: 60%~70% maxHF<br>T: 45 min/session for 6 months<br>T: aerobic exercise on treadmill	**Keep up usual lifestyle**	• Physical therapist<br>• Indoor treadmill<br>• Fully supervised
Niu et al.2020 [33]	China (Taiwan)	60	I:26.07±4.03<br>C:26.03±4.36	I:30(100)<br>C:30(100)	CPSQI>5	2 arms	**Aerobic exercise**<br>F: 3x/week<br>I: 60%~80% maxHF<br>T: 60min/session for 8 weeks<br>T: walking or jogging	**Keep up usual lifestyle**	• Rehabilitation instructor<br>• Indoor treadmill<br>• Fully supervised
Eshaghi et al.2020 [34]	Iran	36	NA	36(100)	PSQI⩾11	4 arms	**Aerobic exercise**<br>F: 3x/week<br>I: 60%~70% maxHF<br>T: 50min/session for 8 weeks<br>T: aerobic training	**Keep up usual lifestyle**	• Exercise physiologists<br>• Laboratory<br>• Fully supervised
Jamshidi et al.2019 [35]	Iran	31	I:63.3±2.5<br>C:65.4±3.2	I:0(0)<br>C:0(0)	PSQI⩾5	2 arms	**Aerobic exercise**<br>F: 3x/week<br>I: 40%~50% maxHF<br>T: 45 min/session for 16 weeks<br>T: aerobic training	**Keep up usual lifestyle**	• NA<br>• NA<br>• Partial supervision with weekly video check-ins
Chen et al. 2019 [36]	China (Taiwan)	40	I:61.0±5.1<br>C:59.8±4.3	I:20(100)<br>C:20(100)	AIS-5>5	2 arms	**Aerobic exercise**<br>F: only once I: 45%~55% max HF<br>T: 50 min session<br>T: walk on a treadmill	**Quiet-rest control**	• Certified instructor<br>• Indoor treadmill<br>• Fully supervised
El-Kader et al. 2019 [37]	Saudi Arabia.	50	I:64.98±4.15<br>C:65.72±3.86	NA	SE<80%, TST<6.5 h awakening before 6 am	2 arms	**Aerobic exercise**<br>F: 3x/week<br>I: 60%~70%, then 70%~80% maxHF<br>T: 40 min/session for 6 months<br>T: Walking/running on treadmill	**Keep up usual lifestyle**	• NA<br>• Indoor treadmill<br>• Fully supervised
Iuliana et al. 2019 [38]	UK	41	59.8±9.46<br>I:59.50±10.59<br>C:60.10±8.51	30(73.17)<br>I：15(75)<br>C：15(71.43)	Research Diagnostic Criteria for insomnia (Edinger et al.,2004)	2 arms	**Aerobic exercise**<br>F: ≥5x/week<br>I: moderate intensity<br>T: ⩾150 min/week, for 6 months<br>T: brisk walking	**wait-list control**<br>Keep up usual lifestyle, and maintain their existing levels of physical activity	• NA<br>• NA<br>• Partial supervision with weekly guidance
Taheri et al. 2018 [39]	Iran	34	＞60	34(100)	PSQI	2 arms	**Aerobic exercise**<br>F: 3x/week<br>I: 50%～60% maxHF<br>T: 60min/session for 2 months T: treadmill or jogging	**Keep up usual lifestyle**	• Exercise physiologist.<br>• Indoor treadmill<br>• Fully supervised

Li-Jung Chen 2016 [40]	China (Taiwan)	67	I:65.2±0.9<br>C:66.2±1.2	I:25(75.0)<br>C:28(82.4)	Actigraphy (SE<85%)	2 arms	**Aerobic/strength exercise**<br>F: 2x/week<br>I: RPE 4–6<br>T: 60 min/session for 8 weeks<br>T: aerobic/strength exercise	**Keep up usual lifestyle**	•Certified aquatic instructor<br>• Indoor swimming pool<br>• Fully supervised
Saba et al. 2016 [41]	Iran	46	I:67.49±4.28<br>C:66.82±3.84	I:0(0)<br>C:0(0)	Primary insomnia based on diagnosis records	2 arms	**Aerobic exercise**<br>F: 3x/week<br>I: walking "as fast as possible"<br>T: 30 min/session for 8 weeks<br>T: walking-based aerobic exercise	**Keep up usual lifestyle**	• Chief nurse / sports medicine specialist<br>•Shahid Yari Elderly House<br>• Fully supervised
Tan et al. 2016 [42]	China	43	51.8±8.4<br>I:51.2(46.6–55.8)<br>C:52.6(48.0–57.2)	I:0(0)<br>C:0(0)	DSM-IV<br>BNSQ	2 arms	**Aerobic exercise**<br>F: 1-5x/week<br>I: 60-75% maxHF<br>T: 30-60min/session for 6 months<br>T: nordic walking or other aerobic training	**Keep up usual lifestyle**	• Experienced exercise trainers<br>• Laboratory<br>• Fully supervised
IULIANA et al. 2015 [43]	UK	41	59.80±9.46<br>I:59.80±9.46<br>C:60.10±8.51	30(73.17)<br>I:15(75)<br>C:15(71.43)	Research Diagnostic Criteria for insomnia (Edinger et al., 2004)	2 arms	**Aerobic exercise**<br>F: 5x/week<br>I: Moderate intensity<br>T: ⩾30 min/session for 6 months T: brisk walking	**Keep up usual lifestyle**	• NA<br>• NA<br>• Monitored program with activity monitor (NewLife NL-1000) and self-reported activity tracking
Jihui et al. 2015 [44]	China	71	I:38.71±9.93<br>C:41.77±7.65	I:22(62.86)<br>C:24(66.67)	ISI ≥10	2 arms	**Aerobic exercise**<br>F: ≥5x/week<br>I: moderate-intensity PA<br>T: 30 min/session for 4 weeks<br>T: walking fast	**Keep up usual lifestyle**	• NA<br>• NA<br>• Fully supervised
Farkhondeh et al. 2015 [45]	Iran	60	64.8±5.2	I:15(50)<br>C:15(50)	PSQI	2 arms	**Aerobic training**<br>F: 3x/week<br>I: Walking at an intensity tolerable for older adults<br>T: 60 min/session for 12 weeks<br>T: walking	**Keep up usual lifestyle**	• NA<br>• NA<br>• Fully supervised
Camila et al. 2024 [46]	Brazil	17	19.3±0.7<br>I:19.09±0.9<br>C:20.0±0.7	11(64.71)	PSQI	2 arms	**Strength training**<br>F: 2x/week<br>I: progressive load<br>T: 60 min/session for 4 weeks<br>T: Resistance training (bench press, pull forward, barbell curl, triceps pulley, leg press 45, extensor, adductor, abductor)	**Control**	• NA<br>• NA<br>• Fully supervised

Samuel et al. 2017 [47]	China (Hong Kong)	216	I:56.6±9.7<br>C:55.6±9.1	I:78(74.3)<br>C:91(82.0)	DSM-IV<br>ICD-10	2 arms	**Strength training**<br>F: 1x/week<br>I: moderate intensity<br>T: 2.5 h/session for 8 weeks<br>T: stretching and muscle strengthening exercises	**MBCT-I**<br>F: 1x/week<br>T: 2.5h/session for 8 weeks	• Experienced physiotherapist<br>• Home<br>• Fully supervised
Jiali et al. 2024 [48]	China (Hong Kong)	38	69.24±4.72<br>I_1:69.85±5.37<br>I_2:68.17±4.47<br>C:69.62±4.46	33（86.84)<br>I_1:11(84.6)<br>I_2:9 (75)<br>C:13(100)	PSQI>5	3 arms	**Flexibility training**<br>F: 3x/week<br>I: moderate intensity<br>T: 60 min/session for 4 weeks<br>T: Simplified Yang-style 12-form Tai Chi	**Treat-as-usual**	• Trained TC instructor.<br>• Community<br>• Fully supervised
Jiali et al. 2024 [49]	China (Hong Kong)	152	67.68±4.98<br>I_1:66.63±5.32<br>I_2:68.50±4.97<br>I_3:67.26±4.84<br>C:68.32±4.73	I_1:26(68.42)<br>I_2:27(71.05)<br>I_3:29(76.32)<br>C:30(78.95)	PSQI>5	4 arms	**Flexibility training**<br>F: 3x/week<br>I: moderate intensity<br>T: 60 min/session for 4 weeks<br>T: Simplified Yang-style 8-form Tai Chi	**Physical exercise**<br>F: 3x/week<br>I: Low intensity<40% HRR<br>T: 60 min/session for 4 weeks<br>T: Aerobic exercise	• Trained instructor<br>• NA<br>• Fully supervised
Siu et al. 2021 [50]	China (Hong Kong)	320	I_1:67.3±5.7<br>I_2:66.5±6.4<br>C:68.0±8.2	I_1:84(80.0)<br>I_2:84(80.0)<br>C:88(80,0)	DSM-5	3 arms	**Flexibility training**<br>F: 3x/week<br>I: moderate intensity<br>T: 1 hour/session, for 12 weeks<br>T: Yang-style 24-form Tai Chi<br>**conventional training**<br>F: 3x/week<br>I: moderate intensity<br>T: 1 hour/session, for 12 weeks<br>T: brisk walking + muscle strengthening exercises	**Routine care**	• Certified instructors<br>• NA<br>• Fully supervised
Judith et al. 2015 [51]	USA	109	I_1:64.8±6<br>I_2:67.3±7.5<br>C:66±7.7	I_1:37(78.7)<br>I_2:25(64.1)<br>C:16(69.6)	DSM-4<br>ICSD-2	3 arms	**Flexibility training**<br>F: 1x/week<br>I: low to moderate intensity<br>T: 120 min/session, for 4 months<br>T: Tai Chi Chuan<br>**CBT**<br>F: 1x/week<br>T: 120 min/session, for 4 months<br>T: behavioral strategies	**Sleep seminar**<br>F: 1x/week<br>T: 120 min/session, for 4 months<br>T: Sleep education	• NA<br>• NA<br>• Fully supervised

Kanika et al. 2023 [52]	India	126	I_1:34.6±7.07<br>I_2:32.15±6.94<br>C:31.8±7.24	I_1:14(35)<br>I_2:18(45)<br>C:15(32.5)	DSM-5	3 arms	**Balance and coordination training**<br>F: 6x/week<br>I: moderate intensity<br>T: 60 min/session, for 4 months<br>T: yoga (physical activity, relaxation, regulated breathing, and philosophical aspects)<br>**ayurveda group**<br>F: morning and evening, daily<br>I: two drops in each nostril<br>T: daily, for 48 days<br>T: Nasya therapy	**Conventional medical treatment**	• Traditional exercise instructors<br>• Outpatient department<br>• Fully supervised
Agustín et al. 2019 [53]	Spain	110	68.18±8.35<br>I:66.79±10.14<br>C:69.98±7.83	I:55（100）<br>C:55（100）	PSQI	2 arms	**Balance and coordination training**<br>F: 2x/week<br>I: Progressive intensity<br>T: 60 min/session, for 12 weeks<br>T: Pilates exercises	**Keep up usual lifestyle**	• NA<br>• NA<br>• Fully supervised
Wing-Fai et al. 2025 [54]	China (Hong Kong)	140	49.9±13.6<br>I:48.0±13.87<br>C:45.3±14.05	I:57(81.4)<br>C:58(82.9)	DSM-5<br>BIQ<br>ISI⩾10	2 arms	**Functional training**<br>F: daily<br>I: NA Low intensity<br>T: 8 weeks<br>T: 10 types of zero-time exercises (simple stretching to resistance training)	**Sleep hygiene education**<br>F: 2x/week<br>T: 2 h/session, for 2 weeks<br>T: sleep hygiene education lessons	• Registered nurse<br>• Home<br>• Fully supervised
Yuan-Gao et al. 2022 [55]	China	118	I:44.3±13.9<br>C:47.7±13.9	I:42(70.0)<br>C:36(62.1)	ICSD-3	2 arms	**Functional training**<br>F: daily<br>I: moderate-intensity<br>T: 3.5 h/day, for 12 weeks<br>T: schedule physical exercise	**Medication treatment**	• NA<br>• Home<br>• Fully supervised
Wing-Fai et al. 2018 [56]	China	37	49.9±13.6<br>I:49.8±13.5<br>C:50.0±14.1	34(91.9)<br>I:16(88.9)<br>C:18(94.7)	DSM-5<br>BIQ<br>ISI⩾10	2 arms	**Functional Training**<br>F: daily<br>I: NA<br>T: 8 weeks<br>T: 10 types of zero-time exercises (simple stretching to resistance training)	**sleep hygiene education**<br>F: 2x/week<br>T: 2 h/session, for 2 weeks<br>T: sleep hygiene education lessons	• Registered nurse<br>• Home<br>• Fully supervised
Glauber Sá et al. 2018 [57]	Brazil	125	I:69.8±7.4<br>C:69.9±6.7	I:56(91.8)<br>C:54(84.4)	PSQI≥5	2 arms	**Functional Training**<br>F: >3x/week<br>I: Borg RPE 13–15<br>T: 40 min/session for 12 months<br>T: aerobic exercises, muscle strengthening, balance, coordination and flexibility	**Sleep hygiene**	• NA<br>• Home<br>• Partial supervision with bi-weekly home assistant
