# Supplementary material for: Physical activity for insomnia: a scoping review within the Nursing Science Precision Health model
Source: Front Public Health. 2026 May 28;14:1834146. doi: 10.3389/fpubh.2026.1834146 (PMC13253286; doi:10.3389/fpubh.2026.1834146)
Supplement: Supplementary file 6 [file Supplementary_File_6.pdf]

Appendix F: Summary of reported sleep-related outcomes in included PA intervention studies

Author/Year	PA modality	Main outcome measured	Intervention-group effect	Between-group effect / difference to control	p value	Direction
Rozales et al. 2024 [26]	Aerobic exercise	***PSG***				
		TST (min)	+50.94 [16.59, 85.30]	+13.44 [−47.45, 74.34]	p > 0.05	TST ↑; between-group^{NS}
		WASO (min)	−24.39 [−49.92, 1.14]	+4.74 [−32.48, 41.95]	p > 0.05	WASO ↓; between-group^{NS}
		SE (%)	+6.61 [0.69, 12.53]	+0.47 [−8.15, 9.10]	p > 0.05	SE ↑; between-group^{N}S
		SOL (min)	−2.33 [−5.40, 0.73]	−0.58 [−5.05, 3.89]	p > 0.05	SOL ↓; between-group^{NS}
		REM latency (%)	+2.44 [−41.87, 46.76]	−58.43 [−123.04, 6.17]	p > 0.05	REM latency ↑; between-group^{NS}
		N1 sleep (%)	−2.22 [−6.63, 2.18]	+0.65 [−5.77, 7.07]	p > 0.05	N1 sleep ↓; between-group^{NS}
		N2 sleep (%)	+4.24 [−2.99, 11.48]	+4.63 [−5.91, 15.18]	p > 0.05	N2 sleep ↑; between-group^{NS}
		N3 sleep (%)	−4.40 [−11.98, 3.18]	−11.92 [−22.97, −0.88]	p = 0.04	N3 sleep ↓; between-group^{S}
		REM sleep (%)	+2.38 [−1.73, 6.49]	+6.68 [0.69, 12.67]	p = 0.03	REM sleep↑; between-group^{S}
		AHI (events/h)	+0.42 [−1.50, 2.35]	+0.02 [−2.78, 2.83]	p > 0.05	AHI ↑; between-group^{NS}
		ArI (events/h)	+1.28 [−4.11, 6.67]	+3.94 [−3.92, 11.80]	p > 0.05	Arousal index ↑; between-group^{NS}
Cammalleri, et al. 2024[27]	Aerobic exercise	***ISI***	−4.7 ± 3.2	NR	Interaction/Time p=0.07/0.005	ISI ↓; time ^{S}, interaction^{NS}
Baron et al. 2023 [28]	Aerobic exercise	***Actigraphy***				
		TST (min)	+32.8 [11.1, 54.5]	+25.7	p = 0.033	TST ↑; between-group^{S}
		WASO (%)	−4.12 [−7.59, −0.65]	−3.48	p = 0.011	WASO ↓; between-group^{S}
		SE (%)	+3.84 [1.12, 6.56]	+2.81	p = 0.222	SE ↑; between-group^{NS}
		SOL (min)	−0.72 [−2.46, 1.02]	−3.09	p = 0.152	SL ↓; between-group^{NS}
Chin et al. 2022 [29]	Aerobic exercise	***Actigraphy***				
		SE (%)	MOD×1/3: +1.5/+1.8<br>VIG×1/3: +0.3/+2.7	MOD×1/3: +1.0/+1.3<br>VIG×1/3: −0.2/+2.2	Interaction/Group/Time p=0.797/0.936/0.419	SE ↑; interaction^{NS}
		WASO (min)	MOD×1/3: −0.3/−2.9<br>VIG×1/3: +0.3/−5.8	MOD×1/3: +4.0/+1.4<br>VIG×1/3: +4.6/−1.5	Interaction/Group/Time p=0.670/0.468/0.458	WASO mixed; interaction^{NS}
		TST (min)	MOD×1/3: +36.5/+59.8<br>VIG×1/3: +81.5/+104.6	MOD×1/3: +27.7/+51.0<br>VIG×1/3: +72.7/+95.8	Interaction/Group/Time p=0.121/0.045/0.614	TST ↑; interaction^{NS}
		SOL(min)	MOD×1/3: −3.4/−0.7<br>VIG×1/3: +1.3/−4.1	MOD×1/3: −3.8/−1.1<br>VIG×1/3: +0.9/−4.5	Interaction/Group/Time p=0.800/0.708/0.510	SOL mixed; interaction^{NS}
Ferreira et al. 2022 [30]	Aerobic exercise	***PSG***				
		TST (min)	+4.4	+40.3	Interaction/Group/Time p > 0.05	TST ↑; interaction^{NS}
		SOL (min)	−4.1	−1.5	Interaction/Group/Time p > 0.05	SOL ↓; interaction^{NS}
		REM latency (min)	−16.7	+57	Interaction/Group/Timep > 0.05/<0 .01/0.04	REM latency ↓; interaction^{NS}
		SE (%)	+3.4	+2.8	Interaction/Group/Time p > 0.05	SE ↑; interaction^{NS}
		WASO (min)	−13.3	-13.9	Interaction/Group/Time p > 0.05	WASO ↓; interaction^{NS}
		N1 sleep (%)	−0.1	−0.1	Interaction/Group/Time p > 0.05	N1 sleep ↓; interaction^{NS}
		N2 sleep (%)	+0.5	−4.6	Interaction/Group/Time p > 0.05	N2 sleep ↑; interaction^{NS}
		N3 sleep (%)	0	−1.3	Interaction/Group/Time p > 0.05	N3 sleep ↔; interaction^{NS}
		REM sleep (%)	−0.4	+7	Interaction/Group/Time p > 0.05	REM sleep ↓; interaction^{NS}
		AHI (events/h)	+2.1	+7	Interaction/Group/Time p > 0.05	AHI ↑; interaction^{NS}

Tseng et al. 2020 [31]	Aerobic exercise	***Actigraphy***				
		SOL (min)	−8.3	−13.2	Interaction/Group/Time p=0.004/0.151/0.694	SOL ↓; interaction^{S}
		SE (%)	+0.04	+0.09	Interaction/Group/Time p=0.088/0.009/0.213	SE ↑; interaction^{NS}
		TST (min)	+19.7	+29	Interaction/Group/Time p=0.271/0.261/0.564	TST ↑; interaction^{NS}
		WASO (min)	+0.4	−21.9	Interaction/Group/Time p=0.500/0.012/0.575	WASO ↑; interaction^{NS}
Abd et al.2020 [32]	Aerobic exercise	***PSG***				
		TST (min)	+27.37	+28.26	p>0.05	TST ↑; between-group^{NS}
		SE (%)	+11.08	+11.86	p<0.05	SE ↑; between-group^{S}
		SOL (min)	+3.59	+3.89	p<0.05	SOL ↑; between-group^{S}
		WASO (min)	−14.88	−19.51	p<0.05	WASO ↓; between-group^{S}
		REM latency (min)	−19.44	−23.16	p<0.05	REM latency ↓; between-group^{S}
Niu et al.2020 [33]	Aerobic exercise	***Actigraphy***				
		TST (min)	T1: +65.02<br>T2: +56.62<br>T3: +40.45	T1: +70.50 [30.42, 109.10]<br>T2: +55.96 [28.92, 83.00]<br>T3: +45.93 [17.32, 74.58]	T1: p<0.001<br>T2: p<0.001<br>T3: p=0.002	TST ↑; between-group^{S}
		SOL (min)	T1: −4.83<br>T2: −4.53<br>T3: −1.82	T1: −7.17 [−13.46, −0.88]<br>T2: −5.59 [−11.84, 0.54]<br>T3: −0.05 [−7.62, 6.61]	T1: p=0.03<br>T2: p=0.08<br>T3: p=0.89	SOL ↓; between-group T1^{S}, T2^{NS}, T3^{NS}
		WASO (min)	T1: −8.45<br>T2: −2.56<br>T3: −2.35	T1: −11.38 [−19.85, −2.91]<br>T2: −3.08 [−13.17, 6.99]<br>T3: −5.43 [−16.58, 5.72]	T1: p=0.008<br>T2: p=0.55<br>T3: p=0.34	WASO ↓; between-group T1^{S}, T2^{NS}, T3^{NS}
		SE (%)	T1: +2.33;<br>T2: +3.23<br>T3: +1.45	T1: +5.20 [2.70, 7.71]<br>T2: +3.96 [1.41, 6.45]<br>T3: +2.78 [−0.42, 5.98]	T1 p<0.001<br>T2 p=0.002<br>T3 p=0.09	SE ↑; between-group T1^{S}, T2^{S}, T3^{NS}
Eshaghi et al.2020 [34]	Aerobic exercise	***PSQI***	−4.67	−4.56	Interaction/Group/Time p<0.001	PSQI ↓; interaction^{S}
Jamshidi et al.2019 [35]	Aerobic exercise	***PSQI***	↓NR	NR	p = 0.001	PSQI ↓; between-group^{S}
Chen et al. 2019 [36]	Aerobic exercise	***Actigraphy***				
		SOL (min)	−3.3 ± 3.6	−4.9	Interaction/Group/Time p<0.001/0.083/0.182	SOL ↓; interaction^{S}
		SE (%)	+3.8 ± 5.7	+5.1	Interaction/Group/Time p=0.025/0.083/0.182	SE ↑; interaction^{S}
		TST (min)	+9.9 ± 35.8	−0.9	Interaction/Group/Time p=0.949/0.820/0.172	TST ↑; interaction^{NS}
		WASO (min)	−10.2 ± 33.9	−20.1	Interaction/Group/Time p=0.069/0.059/0.976	WASO ↓; interaction^{NS}
		NWAK (count)	−1.2 ± 4.4	−0.9	Interaction/Group/Time p=0.482/0.065/0.276	NWAK ↓; interaction^{NS}
		AAT (min)	−0.4 ± 1.4	−0.8	Interaction/Group/Time p=0.133/0.375/0.995	AAT ↓; interaction^{NS}
		TIB (min)	−4.3 ± 39.7	−23.8	Interaction/Group/Time p=0.115/0.195/0.308	TIB ↓; interaction^{NS}
		Activity counts	−5986.7 ± 18630.8	−7249.1	Interaction/Group/Time p=0.285/0.236/0.484	Total counts ↓; interaction^{NS}
El-Kader et al. 2019 [37]	Aerobic exercise	***PSG***				
		TST (min)	+16.54	+18.16	p=0.004	TST ↑; between-group^{S}
		SE (%)	+8.93	+10.06	p=0.006	SE ↑; between-group^{S}
		SOL (min)	+2.27	+2.38	p=0.012	SOL ↑; between-group^{S}
		WASO (min)	−14.12	−13.84	p=0.008	WASO ↓; between-group^{S}
		REM sleep (min)	−15.14	−14.44	p=0.007	REM sleep ↓; between-group^{S}
Iuliana et al. 2019 [38]	Aerobic exercise	***ISI***	−4.06 ± 4.99	−2.64	p=0.03	ISI↓; between-group^{S}

Taheri et al. 2018 [39]	Aerobic exercise	***PSQI***	−8.86	−6.60	p=0.36	PSQI↓; between-group^{NS}
		Sleep quality (score)	−2.00	−2.14	p=0.43	Sleep quality ↑; between-group^{NS}
		Sleep latency (score)	−0.13	0.00	p=0.11	Sleep latency ↑; between-group^{NS}
		Sleep duration (score)	−1.40	−1.40	p=0.99	Sleep duration ↑; between-group^{NS}
		Sleep efficiency (score)	−0.93	−1.26	p=0.36	Sleep efficiency ↑; between-group^{NS}
		Sleep disturbance (score)	−1.26	−1.19	p=0.53	Sleep disturbance ↓; between-group^{NS}
		Medication use (score)	−1.53	−1.53	p=0.99	Medication use ↓; between-group^{NS}
Li-Jung Chen 2016 [40]	Aerobic/ strength exercise	***Actigraphy***				
		SOL (min)	−1.3	−7.9	Interaction/Group/Time p=0.011/0.009/0.401	SOL ↓; interaction S
		SE (%)	+6.7	+5.9	Interaction/Group/Time p<0.001/0.083/0.001	SE ↑; interaction^{S}
		TST (min)	+12.1	+1.4	Interaction/Group/Time p=0.984/0.904/0.207	TST ↑; interaction^{NS}
		WASO (min)	−6.5	−13.7	Interaction/Group/Time p=0.279/0.274/0.864	WASO ↓; interaction^{NS}
		Activity counts	−1749.4	−2717.5	Interaction/Group/Time p=0.680/0.190/=0.906	Activity counts ↓; interaction^{NS}
		NWAK (count)	+2.0	+0.8	Interaction/Group/Time p=0.728/0.923/0.094	NWAK ↑; interaction^{NS}
		AAT (min)	−0.2	+0.7	Interaction/Group/Time p=0.184/0.507/0.023	AAT ↓; interaction^{NS}
Saba et al. 2016 [41]	Aerobic exercise	***PSQI***	−3.5	−4.41	p=0.004	PSQI ↓; between-group^{S}
		Sleep quality (score)	−0.4	−0.13	p=0.03	Sleep quality ↑; between-group^{S}
		Sleep latency (score)	−0.92	−0.49	p=0.001	Sleep latency ↓; between-group^{S}
		Sleep duration (score)	−0.74	−0.13	p=0.006	Sleep duration ↑; between-group^{S}
		Sleep efficiency (score)	−0.44	+0.11	p=0.001	Sleep efficiency ↑; between-group^{S}
		Sleep disturbance (score)	−0.39	−0.19	p=0.009	Sleep disturbance ↓; between-group^{S}
		Medication use (score)	−0.4	−0.45	p=0.043	Medication use ↓; between-group^{S}
		Daytime dysfunction (score)	−0.21	−0.13	p=0.01	Daytime dysfunction ↓; between-group^{S}
Tan et al. 2016 [42]	Aerobic exercise	***Sleep monitoring***				
		TST (min)	+13.3	−6.2	p=0.353	TST ↑; between-group^{NS}
		SOL (min)	−10.2	−12.8	p=0.010	SOL ↓; between-group^{S}
		WASO (min)	−9.2	−3.1	p=0.326	WASO ↓; between-group^{NS}
		SE (%)	+5.4	+4.7	p=0.110	SE ↑; between-group^{NS}
IULIANA et al. 2015 [43]	Aerobic exercise	***ISI***	−4.06 ± 4.99	2.64	p=0.03	ISI ↓; between-group^{S}
Jihui et al. 2015 [44]	Aerobic exercise	***ISI***	−3.51 ± 2.95	−0.22	p=0.749	ISI ↓; between-group^{NS}
		Sleep diary				
		SOL (min)	−19.80 ± 27.34	−9.28	p=0.089	SOL ↓; between-group^{NS}
		TST (min)	+29.83 ± 68.41	+9.14	p=0.168	TST ↑; between-group^{NS}
		TIB (min)	−82.50 ± 50.99	−14.27	p=0.144	TIB ↓; between-group^{NS}
		SE (%)	+0.20 ± 0.12	+0.07	p=0.024	SE ↑; between-group^{S}
Farkhondeh et	Aerobic	***PSQI***	−6.37 ± 2.40	NR	p<0.0001	PSQI ↓; between-group^{S}

al. 2015 [45]	training	sleep duration (hours)	+0.19 ± 0.81	NR	p=0.038	Duration ↑; between-group[S]
		SOL (minutes)	−7.50 ± 16.64	NR	p=0.089	SOL ↓; between-group[NS]
Camila et al. 2024 [46]	Strength training	***PSQI***	−1.0	−1.0	p>0.05	PSQI ↓; between-group[NS]
		SE (%)	−1.9	−4.0	p>0.05	SE ↓; between-group[NS]
		Sleep duration (h)	+0.2	−0.6	p>0.05	Sleep duration ↑;between-group[NS]
		Insomnia score	−1.1	−3.3	p>0.05	Insomnia score↓; between-group[NS]
Samuel et al. 2017 [47]	Strength training	***ISI***	T1: −4.0 ± 3.7<br>T2: −4.4 ± 4.0<br>T3: −5.2 ± 4.1	T1: −0.360 [−0.675, −0.046]<br>T2: −0.179 [−0.515, 0.156]<br>T3: −0.161 [−0.501, 0.180]	T1: p = 0.023<br>T2: p = 0.344<br>T3: p = 0.405	ISI ↓; between-group T1[S], T2[NS],T3[NS]
Jiali et al. 2024 [48]	Flexibility training	***ISI***	T1: −6.31<br>T2: −6.2	T1: −4.62<br>T2: −5.89	Interaction/Group/Time p<0.001/0.003/< 0.001	ISI ↓; interaction[S]
		Actigraphy				
		Sleep efficiency (%)	T1: +0.21; T2: −0.05	T1: +1.63; T2: +1.48	Interaction/Group/Time p=0.015/0.615/0.908	SE mixed; interaction[S]
		TST (min)	T1: −18.79; T2: −11.59	T1: +33.69; T2: +12.12	Interaction/Group/Time p=0.004/0.103/0.141	TST ↓; interaction[S]
		WASO (min)	T1: −4.3; T2: −2.84	T1: −1.34; T2: −1.42	Interaction/Group/Time p=0.594/0.976/0.439	WASO ↓; interaction[NS]
Jiali et al. 2024 [49]	Flexibility training	***ISI***	T1: −3.44; T2: −5.03	T1: −1.39; T2: −2.59	Interaction/Group/Time p=0.343/0.5633/<0.001	ISI ↓; interaction[NS]
Siu et al. 2021 [50]	Flexibility training	***Actigraphy***				
		SE (%)	T1: +1.8; T2: +2.5	T1: +4.7; T2: +7.7	Interaction/Group/Time p<0.001/0.03/0.001	SE ↑; interaction[S]
		WASO (min)	T1: −5.8; T2: −11.1	T1: −18.9; T2: −34.6	Interaction/Group/Time p<0.001/0.02/0.001	WASO ↓; interaction[S]
		NWAK (count)	T1: −2.6; T2: −2.4	T1: −1.4; T2: −2.3	Interaction/Group/Time p<0.001/0.01/0.01	NWAK ↓; interaction[S]
		SOL (min)	T1: +0.5; T2: −0.7	T1: −2.6; T2: −4.9	Interaction/Group/Time p=0.006/0.28/0.003	SOL mixed; interaction[S]
		TST (min)	T1: +8.1; T2: −14	T1: +24; T2: +25.1	Interaction/Group/Time p=0.71/0.58/0.3	TST mixed; interaction[NS]
		AAT(min)	T1: +0.7; T2: +1.1	T1: −0.7; T2: −0.2	Interaction/Group/Time p=0.44/0.2/0.62	AAT ↑; interaction[NS]
Judith et al. 2015 [51]	Flexibility training	***PSQI***	T1: ↔NR; T2: ↓NR	NR	T1: p>0.05<br>T2: p<0.05	PSQI ↓; between-group T1[NS], T2[S]
Kanika et al. 2023 [52]	Balance and coordination training	***PSQI***	4.63 ± 2.28	−7.23 [−9.79, −4.68]	p<0.05	PSQI ↓; between-group[S]
Agustín et al. 2019 [53]	Balance and coordination training	***PSQI***	−1.4	−1.22	Interaction/Group/Time p<0.001/0.667/0.854	PSQI ↓; interaction[S]
		Sleep quality (score)	−0.1	+0.07	Interaction/Group/Time p=0.001/0.161/0.937	Sleep quality ↑; interaction[S]
		Sleep latency (score)	−0.19	−0.29	Interaction/Group/Time p<0.001/0.924/0.661	Sleep latency ↓; interaction[S]
		Sleep duration (score)	−0.38	−0.54	Interaction/Group/Time p<0.001/0.495/0.296	Sleep duration ↑; interaction[S]
		Sleep efficiency (score)	−0.19	−0.11	Interaction/Group/Time p<0.001/0.346/0.774	Sleep efficiency ↑; interaction[S]
		Sleep disturbance (score)	−0.13	−0.42	Interaction/Group/Time p<0.001/0.01/0.342	Sleep disturbance ↓; interaction[S]
		Medication use (score)	−0.22	+0.15	Interaction/Group/Time p<0.001/0.1/0.612	Medication use ↓; interaction[S]
		Daytime dysfunction (score)	−0.14	−0.08	Interaction/Group/Time p=0.008/0.867/0.214	Daytime dysfunction ↓; interaction[S]
Wing-Fai et al. 2025 [54]	Functional training	***ISI***	T1: −4.29<br>T2: −4.49<br>T3: −5.3	T1: −1.23 [−2.54, 0.08]<br>T2: −1.59 [−3.09, −0.08]<br>T3: −2.59 [−4.17, −1.01]	T1: P=0.07<br>T2: P=0.04<br>T3: P=0.001	ISI ↓; between-group T1[NS], T2[S], T3[S]

		Actigraph				
		SOL (min)	T1: +0.89<br>T2: −0.15<br>T3: +0.98	T1: 0.55 [−4.15, 5.26]<br>T2: 3.11 [−1.57, 7.80]<br>T3: 2.80 [−1.99, 7.60]	T1: P=0.82<br>T2: P=0.19<br>T3: P=0.25	SOL mixed; between-group^{NS}
		WASO (min)	T1: −0.59<br>T2: −4.02<br>T3: −4.6	T1: −1.62 [9.07, 5.82]<br>T2: 3.28 [−5.03, 11.60]<br>T3: 1.38 [−7.24, 10.01]	T1: P=0.67<br>T2: P=0.44<br>T3: P=0.75	WASO ↓; between-group^{NS}
		TST (min)	T1: −21.09<br>T2: −12.8<br>T3: −21.53	T1: −13.18 [−31.53, 5.17]<br>T2: −7.68 [−27.66, 12.30]<br>T3: −10.69 [−31.28, 9.90]	T1: P=0.16<br>T2: P=0.45<br>T3: P=0.31	TST ↓; between-group^{NS}
		SE (%)	T1: +0.07<br>T2: +0.16<br>T3: −0.13	T1: −0.46 [−2.34, 1.43]<br>T2: −1.87 [−4.03, 0.28]<br>T3: −1.51 [−3.79, 0.76]	T1: P=0.63<br>T2: P=0.09<br>T3: P=0.19	SE mixed; between-group^{NS}
Yuan-Gao et al. 2022 [55]	Functional training	***PSQI***	T1: −3.02; T2: −7.74<br>T3: −13.06; T4: −15.78	T1: +0.16; T2: −1.36<br>T3: −1.99; T4: −2.28	P < 0.05	PSQI ↓; between-group^{S}
		ISI	T1: −3.86; T2: −6.25<br>T3: −9.34; T4: −11.74	T1: −0.59; T2: −1.86<br>T3: −2.74; T4: −2.61	P < 0.001	ISI ↓; between-group^{S}
		ESS	T1: −2.05; T2: −4.17<br>T3: −5.79; T4: −6.69	T1: −0.64; T2: −1.36<br>T3: −1.34; T4: −1.45	P > 0.05	ESS ↓; between-group^{NS}
Wing-Fai et al. 2018 [56]	Functional Training	***ISI***	T1: −5.2; T2: −5.5<br>T3: −6.2; T4: −6.4	T1: −4.4; T2: −5.3<br>T3: −5.3; T4: −4.6	T1: P=0.001; T2: P=0.002<br>T3: P=0.008; T4: P=0.03	ISI ↓; between-group^{S}
		Actigraphy				
		SOL (min)	T1: −19.6; T2: −26.6	T1: −17.9; T2: −28.9	T1: P=0.56; T2: P=0.18	SOL ↓; between-group^{NS}
		WASO (min)	T1: −14.9; T2: −18.8	T1: −1.4; T2: −8.9	T1: P=0.53; T2: P=0.98	WASO ↓; between-group^{NS}
		TST (min)	T1: +19.4; T2: +28.4	T1: +28.3; T2: +19	T1: P=0.59; T2: P=0.91	TST ↑; between-group^{NS}
		SE (%)	T1: +2; T2: +6.2	T1: +3.1; T2: +3.6	T1: P=0.43; T2: P=0.56	SE ↑; between-group^{NS}
Glauber Sá et al. 2018 [57]	Functional Training	***PSQI***	−4.9 ± 2.7	NR	IG: p < 0.001; CG: p = 0.061	PSQI ↓; within-group IG^{S},CG^{NS}
		Sleep quality (score)	−0.7	−0.7	IG: p < 0.01; CG: p = 0.16	Sleep quality ↑; within-group IG^{S},CG^{NS}
		Sleep latency (score)	−0.9	−0.7	IG: p < 0.01; CG: p = 0.08	Sleep latency ↓; within-group IG^{S},CG^{NS}
		Sleep duration (score)	−0.9	−0.4	IG: p < 0.01; CG: p = 0.11	Sleep duration ↑; within-group IG^{S},CG^{NS}
		Sleep efficiency (score)	−0.9	−0.9	IG: p < 0.01; CG: p = 0.06	SE ↑; within-group IG^{S},CG^{NS}
		Sleep disturbance (score)	−0.7	−0.7	IG: p < 0.01; CG: p = 0.41	Sleep disturbance↓; within-group IG^{S},CG^{NS}
		Medication use (score)	−0.1	−0.1	IG: p = 0.04; CG: p = 0.32	Medication use ↓; within-group IG^{S},CG^{NS}
		Daytime dysfunction (score)	−0.6	−0.6	IG: p < 0.01; CG: p = 0.57	Daytime dysfunction ↓; within-group IG^{S},CG^{NS}
		ESS	−2.8 ± 2.2	NR	IG: p < 0.001; CG: p = 0.63	ESS ↓; within-group IG^{S},CG^{NS}

Note. Values are reported as extracted from the original studies. Intervention-group effect refers to within-intervention change from baseline unless otherwise specified. Between-group effect refers to intervention minus control, group-by-time interaction, or the reported comparative estimate in the original study. Positive values indicate an increase and negative values indicate a decrease. For PSQI component scores, lower scores indicate better sleep. For TST and SE, higher values indicate improvement; for SOL, WASO, ISI, PSQI, ESS, NWAK, and AAT, lower values generally indicate improvement. S indicates statistically significant; NS indicates not statistically significant. AAT, average awaken time; AHI, apnea–hypopnea index; ArI, arousal index; CG, control group; ESS, Epworth Sleepiness Scale; IG, intervention group; ISI, Insomnia Severity Index; MOD, moderate-intensity exercise; NR, not reported; NWAK, number of awakenings; PA, physical activity; PSG, polysomnography; PSQI, Pittsburgh Sleep Quality Index; REM, rapid eye movement; SE, sleep efficiency; SOL, sleep onset latency; T1–T4, study-specific post-baseline or follow-up time points; TIB, time in bed; TST, total sleep time; VIG, vigorous-intensity exercise; WASO, wake after sleep onset.
